# Sorting through the extensive and confusing roles of sortilin in metabolic disease

**DOI:** 10.1016/j.jlr.2022.100243

**Published:** 2022-06-18

**Authors:** Kelly A. Mitok, Mark P. Keller, Alan D. Attie

**Affiliations:** Department of Biochemistry, University of Wisconsin-Madison, Madison, WI, USA

**Keywords:** cholesterol/metabolism, cholesterol/trafficking, dyslipidemias, LDL/metabolism, lipoproteins/metabolism, sortilin, *SORT1*, cellular trafficking, CVD, VPS10, AAV, adeno-associated virus, ADAM, a disintegrin and metalloprotease, AP, adaptor protein, apoB-100, apolipoprotein B-100, ATF3, cyclic adenosine monophosphate transcription factor 3, CAD, coronary artery disease, CD, chow diet, C/EBPα, CCAAT-enhancer-binding protein alpha, *CELSR2*, cadherin EGF LAG seven-pass G-type receptor 2, CES1, carboxylesterase 1, CI, cation-independent, CRE, C-rich element, DLK1, delta-like noncanonical Notch ligand 1, ER, endoplasmic reticulum, EV, extracellular vesicle, FCR, fractional catabolic rate, GGA, Golgi-localized, γ-adaptin ear-containing ADP-ribosylation factor-binding protein, GLUT4, glucose transporter 4, GSV, GLUT4 storage vesicle, GWAS, genome-wide association study, HF/HC, high-fat/high-cholesterol diet, HFD, high-fat diet, IL-6, interleukin 6, LDLR, LDL receptor, MPR, mannose 6-phosphate receptor, mTORC1, mammalian target of rapamycin complex 1, NTR1, neurotensin receptor 1, PCBP, poly-rC-binding protein, PI3K, phosphoinositide-3-kinase, PKC, protein kinase C, p75NTR, p75 neurotrophin receptor, proBDNF, pro-brain derived neurotrophic factor, proNGF, pro-nerve growth factor, PSRC1, proline- and serine-rich coiled-coil 1, PVC, prevacuolar endosome compartment, RAP, receptor-associated protein, SMC, smooth muscle cell, TC, total cholesterol, TG, triglyceride, Tg, transgenic, TGN, *trans*-Golgi network, UTR, untranslated region, VPS10, vacuolar protein sorting 10, VSMC, vascular smooth muscle cell, WAT, white adipose tissue, WD, Western diet, WT, wild-type

## Abstract

Sortilin is a post-Golgi trafficking receptor homologous to the yeast vacuolar protein sorting receptor 10 (VPS10). The VPS10 motif on sortilin is a 10-bladed β-propeller structure capable of binding more than 50 proteins, covering a wide range of biological functions including lipid and lipoprotein metabolism, neuronal growth and death, inflammation, and lysosomal degradation. Sortilin has a complex cellular trafficking itinerary, where it functions as a receptor in the *trans*-Golgi network, endosomes, secretory vesicles, multivesicular bodies, and at the cell surface. In addition, sortilin is associated with hypercholesterolemia, Alzheimer’s disease, prion diseases, Parkinson’s disease, and inflammation syndromes. The 1p13.3 locus containing *SORT1*, the gene encoding sortilin, carries the strongest association with LDL-C of all loci in human genome-wide association studies. However, the mechanism by which sortilin influences LDL-C is unclear. Here, we review the role sortilin plays in cardiovascular and metabolic diseases and describe in detail the large and often contradictory literature on the role of sortilin in the regulation of LDL-C levels.

Sortilin (*SORT1*) was first purified and cloned by affinity chromatography of membrane protein extracts from human brain using receptor-associated protein (RAP) as bait (1). RAP is an endoplasmic reticulum (ER)/Golgi-localized molecular chaperone involved in the folding and processing of members of the LDL receptor (LDLR) family. By binding to these receptors, RAP prevents premature binding of ligands (2, 3, 4, 5). Sortilin was the first receptor not seemingly related to the LDLR family that was found to bind to RAP. Sortilin instead is homologous with yeast vacuolar protein sorting 10 (VPS10) and the cation-dependent and cation-independent mannose 6-phosphate receptors (CD-MPR and CI-MPR), which traffic newly synthesized lysosomal enzymes toward the lysosome. Indeed, soon after its discovery, sortilin was shown to transport several resident lysosomal enzymes to the lysosome (6, 7, 8) as well as traffic other proteins for lysosomal degradation (9, 10, 11, 12, 13).

The initial observation that sortilin binds to RAP suggested that it may be involved in lipoprotein trafficking with the cell. Evidence for such a role came from human genetics. Four genome-wide association studies (GWASs), all published in the same year, found several noncoding SNPs in linkage disequilibrium located at an intergenic region on chromosome 1p13.3 that are strongly associated with circulating LDL-C levels (14, 15, 16, 17, 18). Three genes are located at this locus: cadherin EGF LAG seven-pass G-type receptor 2 (*CELSR2*), proline- and serine-rich coiled-coil 1 (*PSRC1*), and *SORT1*. Follow-up analysis of the key SNP rs646776 revealed that it impacts the mRNA expression of all three genes in human liver, with the largest regulatory effect on *SORT1* mRNA (14).

The GWAS findings led to attempts by many groups to reveal the molecular mechanism behind the association of hepatic *SORT1* expression with LDL-C. Studies in cell lines and mouse model systems have led to contradictory results on the role of sortilin in cholesterol metabolism, the most notable regarding the directionality of the effect of sortilin on apolipoprotein B-100 (apoB-100) trafficking and VLDL secretion in hepatocytes. Studies by Musunuru *et al.* (19) and Kjolby *et al.* (20) found that sortilin regulates VLDL secretion from hepatocytes, thereby affecting LDL-C levels, as VLDL is the precursor of LDL. However, the data published by Musunuru *et al.* (19) showed sortilin to be a negative regulator of VLDL secretion by trafficking the apoB-100-containing lipoprotein toward the lysosome for degradation, whereas Kjolby *et al.* (20) showed sortilin to be a positive regulator of VLDL secretion by trafficking it toward the plasma membrane. These two articles were the foundation for a multitude of studies from several groups, but the reason for the discrepant results is still unknown.

In addition to its role in CVD, a large body of work has established sortilin as a regulator of neuronal development and maintenance and in the pathogenesis of neurological and mood disorders, including Alzheimer’s disease, frontotemporal lobar degeneration, Parkinson’s disease, depression, and anxiety (please see refs. 21, 22, 23, 24 for excellent reviews on this topic).

This review is divided into two major sections. The first revisits fundamental aspects of sortilin’s structure and function, including the tissue distribution and regulation of its expression, the cellular pathways by which it traffics, and its known ligands. The second half reviews and discusses the role that sortilin plays in cardiovascular and metabolic disease, including its involvement in lipoprotein and cholesterol metabolism, and its potential as a drug target. The primary goal of this review is to pull together the more well-recognized (and controversial) ways in which sortilin influences cholesterol metabolism with ones that may have been overshadowed, to better understand the complexity of sortilin’s function.

## Structure and function of sortilin

**Fig. 1:**
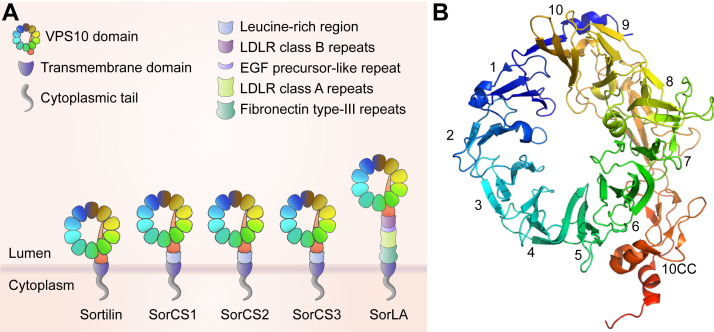
Sortilin is a member of the VPS10 family. A: Sortilin is a member of the mammalian VPS10 family of receptors along with SorLA, SorCS1, SorCS2, and SorCS3, which have a large luminal/extracellular segment containing a VPS10 domain, a transmembrane domain, and a short cytoplasmic/intracellular tail. Diagram adapted from Malik and Willnow (21). B: The VPS10 domain folds into a 10-bladed β-propeller and cysteine-rich 10CC module, as determined by Quistgaard *et al.* (25). Protein Data Bank ID: 3F6K.

Sortilin (encoded by the *SORT1* gene) is a ∼100 kDa type I transmembrane protein and member of the mammalian VPS10 family of post-Golgi trafficking receptors (Fig. 1A) (26, 27). The defining feature of this protein family is the presence of a ∼700 amino acid luminal/extracellular VPS10 domain, which folds into three structural domains: a large N-terminal 10-bladed β-propeller structure and two small C-terminal cysteine-rich domains (together designated the “ten cysteine consensus” or 10CC module) (25) (Fig. 1B). Following the luminal domain, each receptor has a transmembrane domain followed by a short cytoplasmic/intracellular tail of 40–60 amino acids.

### Tissue distribution

Sortilin is expressed in a variety of tissues and cell types. In adult humans, it is highly expressed in tissues like the brain, spinal cord, heart, and skeletal muscle, and lowly expressed in the liver, kidney, pancreas, spleen, and small intestine (1). In the adult human brain, it is predominantly expressed in neurons with regional and neuronal cell-type variability (28). In adult C57BL/6 (B6) mice, sortilin is highly expressed in the hypothalamus, brain, and white adipose tissue (WAT) and lowly expressed in liver and skeletal muscle. In several tissues in mice, including lung, kidney, and pancreas, it is highly expressed during development and then downregulated in adulthood (29). There is a high differential expression in the central nervous system during embryonal development in mice (30, 31). Sortilin is also expressed in immune cells (32, 33, 34, 35).

### Regulation of sortilin expression

Sortilin (*SORT1*) expression is tightly regulated at the transcriptional, post-transcriptional, and post-translational levels by many DNA and RNA binding proteins and signaling pathways in a cell- and tissue-specific manner.

#### Transcriptional regulation

At the DNA level, sortilin expression is regulated in a tissue-specific manner by the transcription factors CCAAT-enhancer-binding protein alpha (C/EBPα), cyclic adenosine monophosphate transcription factor 3 (ATF3), peroxisome proliferator-activated receptor gamma, and signal transducer and activator of transcription 1, and by DNA methylation. Human GWAS have identified SNPs near the *SORT1* gene, located in a noncoding region between the two neighboring genes *CELSR2* and *PSRC1*, which affect the expression of *SORT1*, *CELSR2*, and *PSRC1* in a tissue-specific manner (14, 19, 36, 37). The association of these SNPs with the expression of multiple genes suggests that variation at this locus may have a regional effect on gene expression. Musunuru *et al.* (19) discovered that the minor allele of rs12740374 increases the expression of *SORT1* by creating a binding site for the C/EBP transcription factors in the liver. Furthermore, forced expression of C/EBPα specifically induced *SORT1* expression in hepatocytes but not embryonic cells or adipocytes.

Obesity in humans is associated with downregulation of sortilin at the mRNA and protein levels in subcutaneous WAT (38) and liver (39). Similarly, sortilin mRNA and protein expression is downregulated in the liver, gonadal WAT, and skeletal muscle in response to high-fat diet-induced obesity and genetic obesity (*ob/ob*) in B6 mice (38, 40), making them a good model system for studying the regulation of sortilin expression in obesity. Overnutrition results in hyperactivation of mammalian target of rapamycin complex 1 (mTORC1) and activation of the ER stress response. Ai *et al.* (40) demonstrated that ATF3, which is rapidly induced by ER stress downstream of phospho-eukaryotic initiation factor 2a, binds to a site in the proximal *Sort1* promoter and acts as a transcriptional repressor in liver and adipose tissue. Obesity induces inflammation and a proinflammatory environment, which activates Toll-like receptors and subsequent nuclear factor kappa-light-chain-enhancer of activated B cells activation and ATF3 transcription. Multiple cytokines that are key inflammatory mediators regulate the expression of *Sort1* mRNA. TNFα controls *Sort1* mRNA expression in adipocytes and skeletal muscle partly through a peroxisome proliferator-activated receptor gamma-dependent mechanism (38). IFN-γ controls hepatic *Sort1* levels through the signal transducer and activator of transcription 1 transcription factor, which is activated and bound to the *Sort1* gene upon IFN-γ treatment, reducing the expression of *Sort1* (41). In addition, in a mouse model that is deficient in regulatory T cells, hepatic *Sort1* mRNA expression is significantly reduced, likely through the coincident dramatic increase in hepatic ATF3 in these mice (42).

#### Post-transcriptional regulation

Sortilin expression is regulated by a variety of mechanisms at the RNA level. A network of RNA-binding proteins, including TAR-DNA binding protein 43 (TDP-43), heterogeneous nuclear ribonucleoprotein L (hnRNP L), polypyrimidine tract-binding protein (PTB), and hnRNP A1/A2, is involved in the proper splicing of *Sort1* mRNA (43, 44, 45, 46). Poly-rC-binding proteins 1 and 2 (PCBP1 and PCBP2) stabilize *Sort1* mRNA by recognizing the C-rich element (CRE) in the 3′ untranslated region (UTR) (47, 48). The nucleotide-binding ability of PCBP1 and PCBP2 is impaired by zinc ions, and alterations in intracellular zinc affect *Sort1* expression. In differentiated PC12 cells, C2C12 myotubes, and rat skeletal muscles, *Sort1* expression is positively regulated by glucose through a post-transcriptional mechanism involving 5′ adenosine monophosphate-activated protein kinase and mTORC1, possibly through enhancement of protein translation (49, 50). In addition, the microRNAs miR-182 and miR378a-3p have been shown to bind to the 3′ UTR of *Sort1* mRNA, decreasing *Sort1* mRNA levels and sortilin protein (51, 52).

#### Post-translational regulation

At the protein level, sortilin expression is regulated by palmitoylation, ubiquitination, and phosphorylation of its cytoplasmic tail. Palmitoylation of cysteine 783 in the tail of sortilin stabilizes sortilin protein (53). Nonpalmitoylated sortilin is ubiquitinated by the “neural precursor cell expressed developmentally downregulated 4” E3 ubiquitin protein ligase (NEDD4) and internalized into the lysosomal compartment via the endosomal sorting complexes required for transport pathway for degradation (54). Sortilin is post-translationally downregulated in the liver and gonadal WAT in obesity (38, 39, 40, 55, 56, 57, 58, 59). Saturated fatty acids downregulate hepatic sortilin protein through activation of ERK, which phosphorylates serine 793 in the cytoplasmic tail of sortilin. This phosphorylation event is followed by ubiquitination of lysine 818 and lysosomal degradation (39, 56). Oxidized LDL activates ERK signaling to downregulate sortilin expression in liver sinusoidal endothelial cells (60). In C2C12 myotubes, saturated fatty acids induce downregulation of sortilin via mechanisms involving protein kinase C (PKC) (61).

Sortilin protein is a target of insulin signaling through the insulin/phosphoinositide-3-kinase/protein kinase B (insulin/PI3K/AKT) signaling cascade, whereby insulin increases sortilin protein expression. In hepatocytes, casein kinase II is activated by insulin signaling and phosphorylates serine 825 in the cytoplasmic tail of sortilin, inducing sortilin expression. Inhibition of PI3K signaling or prevention of sortilin phosphorylation induces the lysosomal degradation of sortilin (58). Hepatic sortilin is also a target of leptin signaling, potentially through the action of leptin to stimulate insulin receptor substrate-mediated PI3K activity (62). Interestingly, the insulin/PI3K/AKT signaling cascade also regulates sortilin protein in adipocytes through an unknown mechanism but not through phosphorylation of serine 825 (57).

There is great interest in the significance of the downregulation of sortilin in WAT and liver in obesity. The role of insulin and inflammatory cytokine signaling in regulating liver, adipose, and skeletal muscle sortilin stability suggests that inflammation and impaired insulin signaling (insulin resistance) contribute to reduced sortilin protein in these tissues in obesity.

### Cellular trafficking itinerary of sortilin

#### Sortilin is a post-Golgi trafficking receptor

Sortilin was the first VPS10 domain-containing mammalian protein to be discovered. The domain was first identified in *Saccharomyces cerevisiae* in the sorting receptor protein known as VPS10. Primarily localized in the late Golgi compartment, VPS10 interacts with soluble vacuolar hydrolases, including carboxypeptidase Y (CPY) and proteinase A (PrA), and traffics them to a prevacuolar endosome compartment (PVC) (63, 64, 65). At the PVC, VPS10 releases its ligand and recycles back to the Golgi apparatus for additional rounds of sorting. The hydrolase continues to the vacuole. VPS10 was recognized as being analogous to the CD-MPR and the CI-MPR in mammalian cells. Newly synthesized lysosomal enzymes acquire a mannose 6-phosphate moiety as they pass through the *cis*-Golgi. MPRs then bind these enzymes in the *trans*-Golgi network (TGN) and traffic them to an endosomal compartment. The lysosomal enzymes dissociate from the MPRs in the endosome, where the enzymes continue to the lysosome and the MPRs recycle back to the TGN. The majority of the MPRs traffic between the TGN and endosomes, but some traffic to the cell surface to internalize extracellular lysosomal enzymes (66, 67). The similarity of sortilin to VPS10 and the MPRs prompted initial studies that investigated the involvement of sortilin in targeting lysosomal enzymes to the lysosome in mammalian cells. As predicted, sortilin was found to traffic between the TGN and endosomes (1, 68, 69, 70, 71, 72, 73), mediating the lysosomal targeting of prosaposin (PSAP) (6, 74, 75, 76, 77, 78, 79, 80), GM2 ganglioside activator protein (GM2AP) (6, 75, 76), acid sphingomyelinase (ASM) (7, 80, 81, 82), and cathepsins D and H (8).

**Fig. 2:**
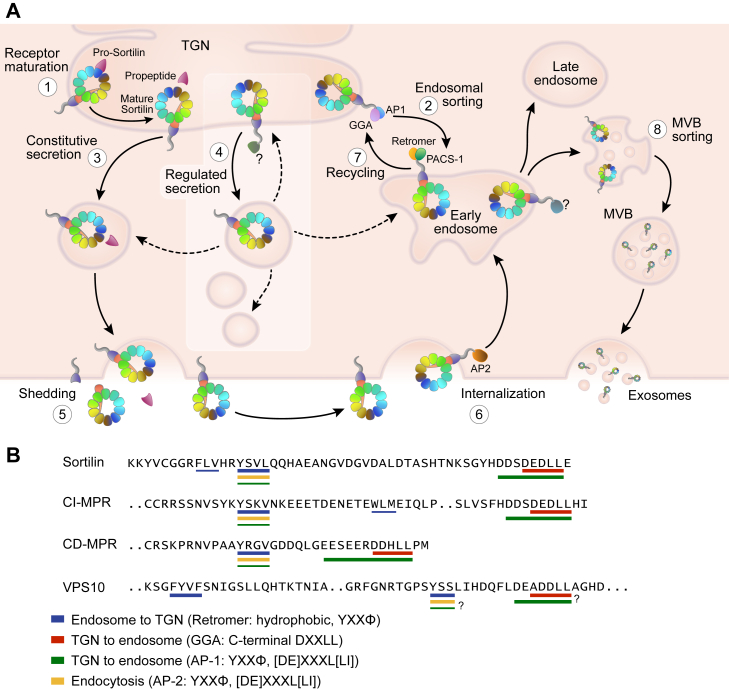
Cellular trafficking of sortilin. A: *1*) Sortilin is converted from its proform to its mature form in the TGN by furin cleavage of its propeptide. Mature sortilin can exit the TGN through three different routes, depending on the cell type, *2*) through anterograde sorting to endosomes, which requires binding of APs GGA and/or AP-1 to its cytoplasmic tail, *3*) through the constitutive secretory pathway, or *4*) through the regulated secretory pathway in specialized cell types. Sortilin does not localize to mature secretory granules, and it is unclear how it exits immature secretory granules, indicated by dotted arrows. When sortilin reaches the cell surface, it can be *5*) shed from the surface, or *6*) endocytosed, which requires AP-2 binding. The majority of sortilin at the plasma membrane is rapidly endocytosed. *7*) Sortilin is returned from endosomes to the TGN by retrograde sorting, which requires interaction with the retromer complex and PACS-1. *8*) Sortilin is also involved in sorting cargo to multivesicular bodies and can itself be secreted from the cell in exosomes. Highlighted box indicates a process that occur in specialized cell types. Diagram adapted from Malik and Willnow *et al.* (21). B: Important sorting motifs located in the cytoplasmic tails of sortilin, CI-MPR, CD-MPR, and yeast VPS10 include a tyrosine-based motif (YXXΦ) and an acidic cluster dileucine motif (DXXLL) that overlaps with an acidic cluster motif ([DE]XXXL[LI]), which are important for AP binding. Thickness of the underline indicates relative potency of the motif for the transport and AP binding indicated. MVB, multivesicular body; PACS-1, phosphofurin acidic cluster sorting protein 1.

Subsequent studies continue to elucidate a much more complex trafficking itinerary of sortilin. In addition to shuttling between the TGN and endosomes, sortilin can also traffic through the constitutive secretory pathway (34), sort into the regulated secretory pathway in specialized cell types (83, 84, 85), function as an endocytosis receptor at the cell surface (11, 68, 86, 87, 88), and aid in exosome formation and release (89, 90, 91, 92) (Fig. 2A).

#### Sorting motifs and adaptor proteins

Like VPS10 and the MPRs, the cytoplasmic tail of sortilin harbors sorting motifs required for the binding of adaptor proteins (APs), Golgi-localized, γ-adaptin ear-containing ADP-ribosylation factor-binding proteins (GGAs), retromer, and other proteins that regulate the trafficking of sortilin (Fig. 2B). Transport of sortilin from the TGN to endosomes is regulated by the binding of GGA1, GGA2, and AP-1 to an acidic cluster dileucine motif (DXXLL, where X is any amino acid) and overlapping acidic cluster motif ([DE]XXXL[LI]) at the far C-terminus of the sortilin tail (68, 69, 71, 93, 94, 95). The dileucine is the most critical part of the motif (68), and it is essential that it be positioned at the C-terminus, as adding extra amino acids to the C-terminus of sortilin (such as a tag) has been shown to inhibit GGA binding (94). AP-1 binding to a tyrosine-based motif in the cytoplasmic tail of sortilin (YXXΦ, where X is any amino acid and Φ is a bulky hydrophobic residue) may also be involved in the TGN-to-endosome transport. At the endosome, the tyrosine-based motif is a potent signal for retromer binding and required for its efficient retrieval back to the TGN for further rounds of sorting (70, 71, 72, 73, 96). There is evidence that a nearby hydrophobic motif, FLV in sortilin and WLM in CI-MPR, may be part of a bipartite retromer binding site (97, 98). This site has similarity to the FYVF site in VPS10 that is required for its retrieval from the PVC in yeast (99). Ceroid-lipofuscinosis neuronal protein 5 (CLN5) has been shown to be required for retromer binding to sortilin at early endosomes (100), and AP-5, a relatively uncharacterized AP, can interact with sortilin and function as a backup to the retromer in retrieving sortilin from the endosome (101). In addition, Rab7b is important for the formation of transport carriers that move sortilin between the TGN and endosomes (102). Calnuc regulates the activity of Rab7 in this process and is also involved in the recruitment of retromer to endosomes (103).

Post-translational modifications of the sorting motifs regulate AP binding, and therefore the trafficking and localization of sortilin. Palmitoylation of cysteine 783 in the cytoplasmic tail of sortilin (nine amino acids N-terminal to the YXXΦ motif) by the aspartic acid-histidine-histidine-cysteine-containing palmitoyltransferase 15 (DHHC-15) is required for efficient retromer binding and retrieval of sortilin from endosomes (53). Palmitoylation is not required for AP-1 binding, suggesting that this modification is required for exit of sortilin from the endosomes but not from the TGN. Mutation of the palmitoylation site results in the trapping of sortilin in endosomes. Unable to recycle, it is subject to ubiquitination and degradation.

The acidic cluster motif in the cytoplasmic tail of sortilin contains a serine residue (serine 825) that can be phosphorylated by casein kinase II (68). Investigation of whether this acidic cluster or the phosphorylation status of its serine residue affects binding of APs and trafficking of sortilin has generated complicated results (68, 69). There is speculation that the hydrophilic nature of the serine residue, but not its phosphorylation status, is important, as has been shown for the sorting of the CI-MPR (104), or that its phosphorylation status is important for binding of GGA2 but not GGA1 (93). Phosphofurin acidic cluster sorting protein 1 (PACS-1) binds phosphorylated acidic clusters, mediating retrograde Golgi-endosome transport (105) and may play a role in the retrieval of sortilin (69, 106). The YXXΦ motif (YSVL) also contains a serine residue, one that can be phosphorylated by Rac-p21-activated kinases 1–3 (107). The phosphorylation of this serine residue alters the affinity for AP-1 binding and changes the intracellular localization of sortilin, supporting prior evidence that the YXXΦ motif is involved in TGN-to-endosome transport through AP-1 binding, in addition to being a potent internalization signal through AP-2 binding at the plasma membrane.

The molecules involved in directing sortilin into the secretory pathways are not as well elucidated. Entry into the regulated secretory pathway requires interaction with still unidentified APs (108). Huntingtin-associated protein 1 (HAP1) may be involved (109). Although it is not an AP itself, it may aid in AP recruitment to the cytoplasmic tail of sortilin. Proteins do not require specific interaction with APs to exit the TGN into the constitutive secretory pathway. It is unknown how sortilin’s entry into this pathway is regulated; however, the “sorting for entry” model of Golgi sorting indicates that proteins enter the constitutive secretory pathway by default if not directly or indirectly bound by APs for regulated secretory pathway or endosome targeting (108). Therefore, sortilin’s entry into the constitutive secretory pathway may be indirectly regulated by post-translational modifications of its cytoplasmic tail that affect binding of APs and entry into these other pathways.

Sortilin molecules that reach the cell surface can have up to three different fates, depending on the cell type. The majority of sortilin receptors at the plasma membrane are rapidly endocytosed. Others become a substrate for a disintegrin and metalloprotease 10 (ADAM10) (10, 110, 111), which cleaves the luminal domain from the transmembrane and cytosolic domains, shedding it from the cell in a soluble form. ADAM17/TNFα-converting enzyme may also be involved in cleaving sortilin (112), but this is controversial and may be cell type specific (10). After the luminal domain is cleaved, the C-terminal fragment left behind in the cell membrane can become a substrate for γ-secretase, potentially aiding in the degradation of the fragment (113). The majority of the cleavage by ADAM10 occurs at the cell surface; however, soluble sortilin has also been detected intracellularly from cleavage by ADAM10 in the secretory pathway, leading to its constitutive secretion from the cell (10). In certain cell types such as neurons, sortilin that reaches the plasma membrane can hetero-oligomerize with the p75 neurotrophin receptor (p75NTR), allowing it to bind pro-nerve growth factor (proNGF) and transmit a signal for apoptosis (see refs. 21, 22, 23, 24 for reviews).

In the steady state, sortilin is predominantly localized to the TGN and endosomes, with a small amount localized (∼10%) at the cell surface (1, 68, 69, 72, 86). At the cell surface, the tyrosine-based motif is a potent signal for internalization by AP-2 (68). The acidic cluster dileucine, to which AP-2 can bind, also plays a role in sortilin internalization, but to a much lesser extent (68). Mutation of the tyrosine-based motif results in the accumulation of sortilin at the plasma membrane (9, 68), indicating that the steady-state localization of sortilin can be deceiving and that a large number of the receptors reach the cell surface but are rapidly internalized.

#### Ligands and binding sites

Sortilin is a multiligand receptor, trafficking and binding a number of soluble and membrane proteins of varying size that have diverse and often unrelated functions. Over 50 different proteins have been identified to bind sortilin and/or have altered trafficking or signaling upon manipulation of sortilin expression or function (Table 1). Neurotensin was the first ligand identified and is the only one that has been co-crystalized with sortilin, revealing its binding in a small pocket inside the tunnel of the 10-bladed β-propeller of sortilin’s VPS10 domain (25). Competitive binding measurements have demonstrated that other ligands, including proNGF, pro-brain derived neurotrophic factor (proBDNF), and progranulin (PRGN) likely bind in a distinct but overlapping region with that of neurotensin (25, 165, 166), revealing that at least part of the binding site of proneurotrophins is located outside the tunnel of the β-propeller (166).

**Table 1:** Sortilin ligands

Pathway	Ligand	References
Lipid related	Apolipoprotein A-V (apoA-V)	(87)
	Apolipoprotein B-100 (apoB-100)	(9, 20, 114, 115, 116)
	Apolipoprotein E (apoE)	(117)
	Apolipoprotein J/clusterin (apoJ)	(118)
	ATP-binding cassette subfamily A member 1 (ABCA1)	(12)
	Delta like non-canonical Notch ligand 1 (DLK1)	(95)
	Lipoprotein lipase (LPL)	(11)
	Carboxylesterase 1 (CES1)	(13)
	Proprotein convertase subtilisin/kexin type 9 (PCSK9)	(119)
Neurotrophin related	p75 neurotrophin receptor (p75NTR)	(120, 121, 122)
	pro-brain-derived neurotrophic factor (proBDNF)	(10, 35, 45, 84, 109, 120, 123, 124)
	pro-nerve growth factor (proNGF)	(121, 125, 126, 127, 128)
	Proneurotrophin-3 (proNT-3)	(129)
	Tropomyosin receptor kinase A (TRKA)	(130)
	Tropomyosin receptor kinase B (TRKB)	(130)
	Tropomyosin receptor kinase C (TRKC)	(130)
Neurotensin related	Neurotensin	(25, 86, 131, 132)
	Neurotensin receptor 1 (NTR1)	(133, 134)
	Neurotensin receptor 2 (NTR2)	(135)
Amyloid precursor protein (APP) related	Amyloid-precursor-like protein 2 (APLP2)	(136)
	Amyloid precursor protein (APP)	(137, 138)
	β-site amyloid precursor protein cleaving enzyme 1 (BACE1)	(139)
Cytokine related	Cardiolipin-like cytokine/cytokine-like factor-1 (CLC/CLF-1)	(140)
	Ciliary neurotrophic factor (CNTF)	(140)
	Glycoprotein 130/leukemia inhibitory factor receptor β (gp130/LIFRβ)	(140)
	Interferon-α (IFN-α)	(48)
	Interferon-γ (IFN-γ)	(33, 34, 48)
	Interleukin-6 (IL-6)	(33, 48)
	Interleukin-10 (IL-10)	(48)
	Interleukin-12 (IL-12)	(48)
	Interleukin-17A (IL-17A)	(48)
	Neuropoietin	(140)
Lysosomal proteins	Acid sphingomyelinase (ASM)	(7, 81)
	Cathepsin D	(8)
	Cathepsin H	(8)
	Prosaposin (PSAP)	(6, 75, 78, 79, 80)
Other	Activin	(141)
	Adiponectin	(142)
	α-galactosidase A (α-Gal A)	(88)
	α-synuclein	(143)
	Bone morphogenic protein 4 (BMP4)	(141)
	Epidermal growth factor receptor (EGFR)	(144, 145)
	Gelsolin	(146)
	Glucose transporter 4 (GLUT4) storage vesicles	(83, 85, 147, 148, 149, 150, 151, 152)
	GM2 ganglioside activator pseudogene (GM2AP)	(6, 75)
	Golgi phosphoprotein 4 (GPP130)	(153)
	Na^+^/Cl^−^ cotransporter (NCC)	(154)
	Phosphatidylinositol (3,4,5)-triphosphate (PIP3)	(155)
	Prion (PrP^C^ and PrP^Sc^)	(156)
	Progranulin (PGRN)	(43, 157, 158)
	Prorenin receptor (PRR)	(159)
	Receptor-associated protein (RAP)	(1)
	Sonic hedgehog (SHH)	(160)
	Thyroglobulin	(161, 162)
	TWIK-related potassium channel 1 (TREK-1)	(163, 164)

A comprehensive list of known ligands or receptor binding partners of sortilin.

#### Regulation of ligand binding and trafficking

Sortilin is synthesized as a proprotein and converted to its mature form in the late Golgi by furin cleavage of its propeptide. The propeptide binds inside the tunnel of the β-propeller with high affinity and inhibits binding of some of sortilin’s ligands in the early secretory pathway, including neurotensin (25, 166, 167) and RAP (167). Interestingly, binding of the propeptide to sortilin does not block the binding of all ligands, including proNGF and proBDNF (166), supporting the view that sortilin has multiple binding sites for ligands. Recently, a small molecule that specifically binds to “binding site 2” (the site where neurotensin does not bind) was shown to augment binding of neurotensin to sortilin binding site 1, suggesting that site 2 is an allosteric regulator of site 1 binding (168).

Since sortilin can bind to multiple ligands and traffic them through several possible pathways in the same cell, various questions emerge: Upon binding of a particular ligand, what determines which of the multiple trafficking pathways are pursued? What is the link between specific ligand binding and recruitment of the appropriate APs to transport a ligand to its correct destination? The answers to these questions are largely unknown. However, recent work by Trabjerg *et al.* (165) using hydrogen/deuterium exchange mass spectrometry found that different ligands exhibit distinct conformational impacts on sortilin. These specific ligand binding-induced conformational changes extend into the membrane-proximal domain of sortilin, and potentially across the membrane, possibly affecting AP binding. This hints at a mechanism by which sortilin mediates diverse ligand-dependent trafficking. Another possibility is that ligands destined for different pathways localize to different regions of the Golgi, prior to their interaction with sorting receptors. Recently, the Bonifacino group provided direct evidence for this additional level of protein sorting in the Golgi, where there is early segregation of different sets of proteins that are destined for different pathways, well before their export in transport carriers (169). This creates regions of the Golgi that generate carriers destined for the constitutive secretory pathway that are distinct from regions that generate carriers destined for the endolysosomal system, for example. Therefore, it is possible that sortilin localized to the section of the Golgi that buds transport carriers destined for the endolysosomal system only has access to proteins that have been presorted for this pathway. Generation of these carriers and their targeting to the endolysosomal pathway would require AP binding to receptors. Similarly, sortilin localized to the section of the Golgi that generates transport carriers destined for the constitutive secretory pathway would only have access to proteins that have been presorted for this pathway. However, these carriers would be generated and targeted independently of APs.

Interestingly, in some cases, sortilin has been shown to traffic the same ligand to different pathways depending on the cellular context. For example, under normal conditions, sortilin targets proBDNF to the regulated secretory pathway in neurons. However, under conditions where the cell has excess proBDNF, sortilin targets this excess to the endolysosomal system for degradation (10, 84). In hepatocytes, sortilin may traffic apoB-100 toward the secretory pathway for secretion or toward the lysosome for degradation, depending on the metabolic context (114, 115, 170). The mechanism underlying these switches is unknown.

**Fig. 3:**
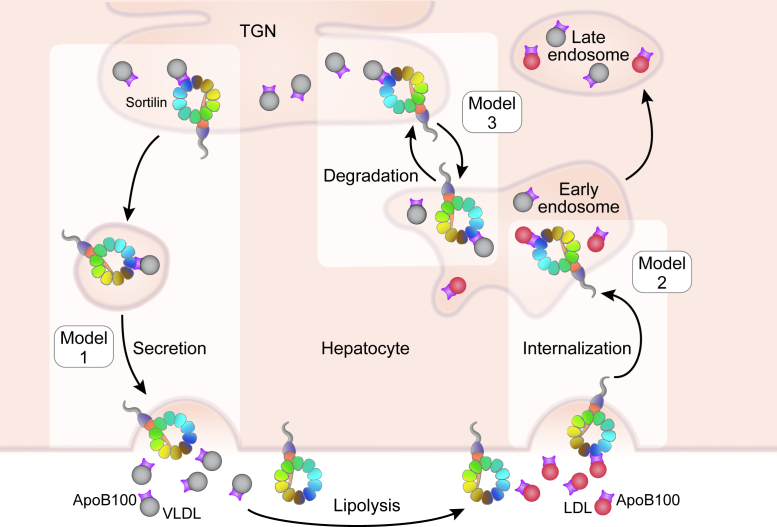
Models of sortilin function in lipoprotein metabolism in the liver. Model 1: Sortilin facilitates the secretion of VLDL, increasing circulating VLDL and LDL through lipolysis. Model 2: Sortilin promotes LDL internalization, decreasing circulating LDL. Model 3: Sortilin traffics VLDL from the TGN toward the endolysosomal system for degradation, decreasing circulating VLDL and LDL. Diagram adapted from Schmidt and Willnow (175). LDL, red shaded; VLDL, gray shaded.

Ligand binding is also regulated by dimerization of sortilin at low pH. During sortilin’s transport between the TGN, cell surface, endosomes, and other vesicular compartments, it is exposed to dramatic fluctuations in pH. Ligands tend to show high affinity for sortilin at neutral pH but have a reduced or a complete loss of affinity at acidic pH (1, 119, 137, 167, 171), consistent with release of ligands in secretory granules or late endosomes. Recent reports by several groups have revealed that low pH triggers sortilin to undergo a conformational change and dimerize, causing the collapse of the binding site in the tunnel of the β-propeller and release of the ligand (92, 172, 173, 174) (Fig. 3). Sortilin is predominantly a monomer at neutral pH and predominantly a dimer at acidic pH. It dimerizes through the top face of its β-propeller, opposite the 10CC module. Hydrophobic loops that protrude from the blades of the β-propeller at the dimer interface are important for dimer formation. In addition, disruption and formation of Coulombic repulsions between charged residues (173), salt bridges (173), and disulfide bonds (92) are important for the conformational changes and monomer-dimer shift that occurs upon pH change. Only structures of the sortilin luminal domain were determined, but the structure of the soluble sortilin dimer reveals that the C termini of the luminal domains are in close proximity to each other, indicating that the 2-fold axis that describes the dimer is oriented perpendicular to the cell surface (173). The ligand binding site located in the tunnel of the β-propeller undergoes a conformational change in the monomer-dimer transition that triggers release of ligand from sortilin (173). Januliene *et al.* (174) proposed the appealing idea that the various cytosolic APs may have different affinities for the cytoplasmic tail of sortilin when in the monomeric or dimeric state. Therefore, dimerization may be a mechanism by which sortilin traffics specific ligands toward different pathways in the cell.

## The role of sortilin in cardiovascular and metabolic disease

Sortilin is implicated in many aspects of health and disease through its function in the cellular trafficking of over 50 different molecules, including apolipoproteins, cytokines, proneurotrophins, and enzymes, and also being a coreceptor for neurotrophin signaling (Table 2). Sortilin is involved in many facets of cardiovascular and metabolic disease pathogenesis, including atherosclerosis, lipoprotein metabolism, vascular calcification, obesity, insulin resistance, and glucose homeostasis. This section reviews the extensive evidence linking sortilin to these pathways and diseases and discusses its potential as a drug target.

**Table 2:** Pleiotropic role of sortilin in disease

Pathway or disease	Mechanism	References
Cardiovascular and metabolic disorders		
Lipoprotein metabolism	Hepatic VLDL trafficking	(9, 19, 20, 39, 40, 114, 115, 116)
	Hepatic LDL clearance	(9, 37, 176)
	Hepatic PCSK9 secretion	(119)
	LPL trafficking	(11)
	ApoA-V trafficking	(87)
	Altered plasma cholesterol, unknown mechanism	(55, 177, 178)
Atherosclerosis	Lipoprotein metabolism (see above)	
	Macrophage proinflammatory cytokine trafficking	(33)
	Macrophage LDL uptake and foam cell formation	(32)
	Macrophage ABCA1 trafficking	(12)
	Smooth muscle cell apoptosis via proNT signaling	(179)
	Osteoblastic differentiation and vascular calcification	(51, 90, 180, 181)
Obesity, insulin resistance, and glucose homeostasis	Adipocyte and myocyte GLUT4 vesicle trafficking	(61, 83, 148, 150, 152)
	Adipocyte differentiation	(95, 182)
	Intestinal lipid absorption via neurotensin binding	(178, 183)
	Altered diet-induced obesity or insulin resistance, unknown mechanism	(55, 59, 178, 184)
Neurological and neurodegenerative disease		
Neuronal development and maintenance	Binds proNGF and p75NTR, forming apoptotic signaling complex in neurons	(121, 127)
	proBDNF signaling and trafficking	(10, 35, 45, 84, 109, 120)
	TRKA, TRKB, and TRKC trafficking	(129)
Alzheimer’s disease	APP and BACE1 trafficking	(137, 138, 139)
	Neuronal apoE and apoJ metabolism	(117, 118)
	Tau prion trafficking	(185)
	proNT signaling and trafficking	(186, 187, 188, 189)
	Aβ toxicity mediated by p75NTR-sortilin complex	(190)
Prion diseases	PrP^c^ and PrP^Sc^ trafficking	(156)
Frontotemporal dementia	Clearance of PGRN	(43, 157, 191, 192)
Parkinson’s disease	p75NTR-sortilin assembly in substantia nigra neurons	(193)
	α-synuclein trafficking	(143)
Depression and anxiety	proNT signaling and trafficking	(194, 195, 196, 197, 198, 199)
	TREK-1 trafficking	(163, 164)
Other		
Cancer	Neurotensin, proNT, and PGRN signaling and trafficking	(134, 144, 145, 200, 201, 202, 203, 204, 205, 206, 207, 208, 209, 210, 211, 212, 213, 214, 215, 216, 217)
	EGFR trafficking	(144, 145)
Immune processes and inflammation	Proinflammatory cytokine and receptor trafficking	(33, 34, 48, 140)
	Microglia activation and migration via neurotensin binding	(218, 219, 220, 221)

List of diseases and pathways that sortilin has been implicated in and the mechanism(s) by which it is involved in the pathology.

### SNPs controlling hepatic *SORT1* expression are associated with LDL-C in human GWAS

Human GWAS have identified several SNPs (rs599834, rs646776, rs629301, rs660240, rs602633, and rs12740374) in a haplotype block in the region of the gene cluster *CELSR2-PSRC1-SORT1* on chromosome 1 at the 1p13.3 locus that strongly associate with LDL-C levels in several cohorts and ethnic populations (14, 15, 16, 17, 36, 222, 223, 224, 225, 226, 227, 228, 229). The minor alleles of these SNPs are protective, associated with a 5–8 mg/dl decrease in LDL-C. The effect size of the association between the locus and LDL-C levels is larger in younger populations and males (230, 231, 232) and is independent of obesity (182). In addition, rs646776 displayed a major impact on statin efficacy to reduce LDL-C levels in an elderly population (233). Of particular relevance, the minor allele of rs646776 has been shown to be most highly associated with levels of small LDL (19). In humans, there are several distinct subclasses of LDLs that range in size and density (234). The small dense LDLs are associated with risk of atherosclerotic CVD, being more atherogenic than their larger and more buoyant counterparts (235, 236, 237, 238, 239). In recent years, an increasing number of studies demonstrating that small dense LDLs have a greater propensity to cause atherosclerosis have emerged. This has propelled researchers to further investigate the mechanism behind the atherogenicity of small dense LDLs in order to develop new therapies to prevent cardiovascular events and to establish a clinically effective method to accurately measure circulating small dense LDL levels (240, 241, 242, 243).

The six LDL-C-associated SNPs at the 1p13.3 locus cluster in a noncoding region that is 6.1 kb in length, spanning the 3′ UTR of *CELSR2*, the intergenic region, and the 3′ UTR of *PSRC1*, and downstream of *SORT1*. These SNPs seem to regulate the expression of *SORT1*, *PSRC1*, and *CELSR2* in a tissue-specific manner. Schadt *et al.* (36) found that the minor allele of rs599839 is associated with increased hepatic *SORT1* and *CELSR2* expression and decreased *PSRC1* expression. Studies by Kathiresan *et al.* (14) and Musunuru *et al.* (19) showed that the minor allele of rs646776 is associated with increased hepatic expression of all three genes with the increase in *SORT1* expression being the largest. Kathiresan *et al.* found that rs646776 explained 86, 58, and 58% of the interindividual variability in *SORT1*, *CELSR2*, and *PSRC1* expression levels, respectively. In analyses conditioning on either the *CELSR2* or *PSRC1* transcript levels, rs646776 remained associated with *SORT1* expression. Conversely, when conditioning on *SORT1* expression, rs646776 was weakly or not associated with *PSRC1* or *CELSR2* expression. In addition, Musunuru *et al.* found that the minor allele of rs12740374 is associated with increased hepatic *SORT1* and *PSRC1* expression and not associated with the expression of *CELSR2* in the liver. By analyzing haplotype maps from humans of varying ethnicity, Musunuru *et al.* identified rs12740374 as the causal SNP in the haplotype block and determined that the minor allele generates a C/EBP transcription factor binding site, increasing *SORT1* expression. There was no association between the SNPs and *SORT1* expression found in studies of WAT (19), whole blood (244), monocytes (245), or blood vessels (246, 247). These analyses suggested that the regulatory mechanism underlying the association of the SNPs at the 1p13.3 locus with LDL-C was sortilin mediated and liver specific and predicted an inverse relationship between hepatic *SORT1* expression and circulating LDL-C level.

### The controversial role of sortilin in hepatic lipoprotein metabolism

With *SORT1* being nominated as the gene responsible for the association of the 1p13.3 locus with LDL-C, functional studies by several groups sought to validate this finding and determine the underlying mechanism. The prevailing conclusion is that sortilin plays a direct role in trafficking apoB-100 containing lipoproteins in hepatocytes. However, the directionality of sortilin’s effects is highly disputed.

#### Sortilin promotes cellular LDL uptake, but is this dependent on the LDLR?

The first evidence linking sortilin function with LDL-C levels showed an effect of sortilin overexpression on cellular LDL uptake. Linsel-Nitschke *et al.* (37) overexpressed *SORT1* in human embryonic kidney 293 cells and found increased internalization of radiolabeled LDL particles. Subsequent studies in HeLa cells (176) and the human hepatocyte cell line HuH7 (9) produced the same result. Conversely, in the HepG2 human hepatocyte (159), A431 human epidermoid carcinoma (159), and HeLa (248) cell lines, silencing of *SORT1* reduced LDL uptake. Importantly, the studies in HeLa, HepG2, and A431 also looked for an effect of *Sort1* manipulation on the total and/or cell surface abundance of LDLR protein. *SORT1* overexpression in HeLa cells had no effect on the amount of LDLR at the cell surface (176). The studies measuring only total cellular abundance of LDLR protein produced conflicting results (136, 159, 248). Measurement of total hepatic LDLR protein abundance in *Sort1*^*−/−*^ mice has also produced conflicting results (119, 136).

To directly assess whether the LDLR is required for the effect of sortilin on LDL uptake, Strong *et al.* (9) measured the clearance of LDL from the circulation in chow diet (CD)-fed female mice with either global genetic deletion or liver-specific overexpression of *Sort1* in both wild-type (WT) and *Ldlr*^*−/−*^ backgrounds. In a WT background, knockout of *Sort1* resulted in a lower fractional catabolic rate (FCR) of radiolabeled LDL, and overexpression caused an increase in the LDL FCR. On an *Ldlr*^*−/−*^ mouse background, *Sort1* overexpression also resulted in increased LDL clearance. The authors state that *Sort1*^*−/−*^*;Ldlr*^*−/−*^ mice, compared with *Ldlr*^*−/−*^ mice, have a 50% lower LDL FCR. However, the curves showing the percent LDL remaining in the circulation over time that were used to calculate the FCRs appear nearly identical (panels E and F in Fig. 4 in ref. 9). Therefore, from this in vivo work in B6 mice, it appears that the LDLR is required for sortilin to promote LDL clearance at a normal physiological level of sortilin, but when expressed at a supraphysiological level, sortilin may promote the clearance of LDL independently of the LDLR, possibly by directly binding and internalizing LDL itself. Interestingly, an article by Patel *et al.* (32) found that bone marrow-derived macrophages isolated from *Sort1*^*−/−*^ mice internalized 40% less LDL in both WT and *Ldlr*^*−/−*^ backgrounds, suggesting that a requirement for the LDLR for sortilin to promote LDL uptake may be dependent on cell type. Contradictory to the findings by Strong *et al.*, a study by Kjolby *et al.* found no difference in the uptake of radiolabeled LDL into primary hepatocytes isolated from WT and *Sort1*^*−/−*^ mice (20). However, primary hepatocytes begin losing mature function within the first few hours of traditional in vitro culture (249, 250, 251), possibly explaining the lack of an effect on loss of *Sort1* on LDL uptake in these experiments.

Any experiments involving overexpression of sortilin need to be interpreted cautiously because both overexpression and C-terminal tagging can cause sortilin to mislocalize and become unphysiologically abundant at the cell surface. The predominant localization of sortilin to the TGN and endosomes is dependent upon interaction of APs, GGAs, and retromer with tyrosine- and dileucine-based sorting motifs in its C-terminus. C-terminal tagging of sortilin inhibits GGA binding (94), and mutation of the tyrosine and dileucine sorting motifs in sortilin’s cytoplasmic tail results in its accumulation at the plasma membrane (9, 68). Furthermore, overexpression of sorting receptors containing functional tyrosine- and dileucine-based sorting signals results in their accumulation at the cell surface because of the saturation of APs (252). This is discussed in more detail in a later section.

The majority of in vitro and in vivo work supports a role for sortilin in promoting LDL clearance by hepatocytes, which would work to lower LDL-C and is therefore consistent with the directionality predicted by the human genetics. The mechanism by which sortilin does this, however, seems to depend on whether it is expressed at a normal level or a supraphysiological level.

#### Sortilin regulates hepatic apoB-100 secretion, but in what direction?

Since circulating LDL levels are determined by both its rate of clearance and rate of production, several groups have also investigated a role for sortilin in the secretion of VLDL from the liver.

Musunuru *et al.* (19) were the first to publish on the effect of hepatic *Sort1* expression on apoB-100 secretion. They treated four different C57BL/6 CD-fed mouse models with an adeno-associated virus 8 vector encoding the murine *Sort1* gene driven by the liver-specific thyroxine binding globulin promoter (AAV8-TBG), resulting in liver-specific overexpression of *Sort1*. In all four backgrounds tested (*Apobec*^*−/−*^; *APOB Tg*, *Apobec*^*−/−*^; *Ldlr*^*−/−*^, *Apobec*^*−/−*^; *APOB Tg*; *Ldlr*^*+/−*^, *and Apobec*^*−/−*^; *APOB Tg*; *Ldlr*^*−/−*^), overexpression of *Sort1* resulted in a significant reduction in plasma total cholesterol (TC) and LDL-C by 23–76% depending on the background. Conversely, knockdown of *Sort1* with liver-targeted *Sort1* siRNA in the same mouse models resulted in a significant increase in plasma TC and LDL-C by 16–125% depending on the background. Similarly, they observed a 50% increase in plasma TC and LDL-C in CD-fed *Sort1*^*−/−*^ compared with WT mice. These findings were concordant with directionality predicted by the human genetics. To determine the mechanism behind this effect, the group assessed the rate of in vivo VLDL secretion by injecting mice with detergent to inhibit the lipolysis of VLDL and subsequently measuring the accumulation of circulating triglyceride (TG) and VLDL (via NMR) over time. *Sort1* overexpression in *Apobec*^*−/−*^*;* *APOB Tg* decreased the rate of VLDL accumulation by 57%. Furthermore, overexpression of *Sort1* in primary hepatocytes isolated from the *Apobec*^*−/−*^*;* *APOB Tg;* *Ldlr*^*+/−*^ or *Apobec*^*−/−*^*;* *Ldlr*^*−/−*^ mice overexpressing *Sort1* significantly decreased the secretion of newly synthesized apoB-100. Consistently, secretion of apoB-100 was significantly increased in hepatocytes isolated from *Apobec*^*−/−*^*;* *APOB Tg;* *Ldlr*^*+/−*^ mice treated with *Sort1* siRNA. From these data, the authors proposed a model in which sortilin negatively regulates hepatic export of VLDL, the precursor of LDL, thereby decreasing the production of LDL from VLDL.

Nearly simultaneously, Kjolby *et al.* (20) published data contradicting that of Musunuru *et al.* In this study, *Sort1*^*−/−*^ mice on a C57BL/6 background and fed a Western diet (WD; 43% kcal from fat, 0.15% cholesterol) for 6 weeks had a 20% reduction in plasma TC and a 65% decrease in LDL-C (20). On an *Ldlr*^*−/−*^ background, *Sort1*^*−/−*^ reduced plasma TC by 30% and plasma LDL-C by 25%. Using adenoviral gene transfer, they overexpressed *Sort1* in the liver of WT mice and found a 42% increase in plasma TC. Similarly, *Sort1* overexpression on the *Ldlr*^*−/−*^ background increased plasma TC by 33% and increased LDL-C, restoring them to the levels of *Ldlr*^*−/−*^ mice. Using a similar detergent-based method as Musunuru *et al.*, they assessed VLDL secretion in WD-fed WT and *Sort1*^*−/−*^ mice but found that *Sort1*^*−/−*^ resulted in slower accumulation of circulating TG and total apoB-100 levels. Consistently, in primary hepatocytes isolated from the *Sort1*^*−/−*^ mice, newly synthesized apoB-100 secretion was decreased by 54%. Using coimmunoprecipitation and surface plasma resonance, they found that sortilin can directly bind apoB-100. From these data, they concluded that sortilin acts as a positive regulator of VLDL export in the liver, increasing VLDL secretion (20).

Shortly thereafter, the Rader group published a second article that presented further evidence of sortilin acting as a negative regulator of VLDL export (9). In agreement with the group’s initial finding, they found that overexpression of *Sort1* via AAV8-TBG in CD-fed female WT and *Ldlr*^*−/−*^ mice reduced newly synthesized VLDL apoB-100 secretion into the circulation by 30% and 50%, respectively. To test if the endolysosomal trafficking of sortilin was required for the reduction in apoB-100 secretion, they conducted similar experiments with two different sortilin mutants that cannot traffic to the lysosome: Sort.LAYA, in which critical residues in the dileucine and tyrosine sorting motifs are mutated to alanine, and Sort.stop, which lacks the entire transmembrane domain and cytoplasmic tail. Overexpression of either mutant in *Ldlr*^*−/−*^ mice failed to reduce apoB-100 secretion. Furthermore, overexpression of *Sort1* in hepatocytes isolated from *Apobec*^*−/−*^; *APOB Tg*; *Ldlr*^*+/−*^ mice resulted in decreased apoB-100 secretion, and this effect was completely inhibited by treatment with the endolysosomal inhibitor E64d. In contrast to their group’s original finding, however, their data showed that *Sort1*^*−/−*^ in an *Apobec*^*−/−*^; *APOB Tg* mouse background had a 60% *decrease* in VLDL apoB-100 secretion. Therefore, in this article, *both* overexpression and knockout of *Sort1* resulted in decreased VLDL apoB-100 secretion. In addition, both Strong *et al.* and a third article from this group by Patel *et al.* (32) reported normal plasma TC and LDL-C levels in *Sort1*^*−/−*^ mice on an *Apobec*^*−/−*^; *APOB Tg* background, in contrast to the increased levels observed by Musunuru *et al.* upon siRNA knockdown of *Sort1* in the same mice.

The story became increasingly confusing and complex as reports from several other groups emerged. Some agreed with the findings of Kjolby *et al.*, showing a positive relationship between *Sort1* level and plasma cholesterol and/or hepatic apoB-100 secretion (12, 55, 178, 184), whereas others observed a negative relationship, in agreement with Musunuru *et al.* (39, 40, 116, 177) (Table 3). Others found *Sort1*^*−/−*^ to have no effect on plasma cholesterol (33, 90, 136) (Table 3).

**Table 3:** Paradoxical role of sortilin in lipoprotein metabolism

Model system	Diet	Sex	Method	TC/LDL-C	apoB secretion	LDL uptake	Reference
Upregulation of Sort1							
HEK293 cells	NA	NA	Plasmid	NA	ND	↑	Linsel-Nitschke *et al.* (37)
HeLa T-Rex cells	NA	NA	Plasmid	NA	ND	↑	Tveten *et al.* (176)
HuH7 cells	NA	NA	LV	NA	↓	↑	Strong *et al.* (9)
McA cells	NA	NA	Plasmid	NA	—	ND	Conlon *et al.* (170)
hAPOB McA cells	NA	NA	Plasmid	NA	↓	ND	Amengual *et al.* (116)
hAPOB McA cells	NA	NA	Plasmid	NA	↓	ND	Conlon *et al.* (170)
HepG2 cells	NA	NA	AV	NA	↓	ND	Bi *et al.* (39)
WT mouse heps	CD	♂	AV	NA	↓	ND	Bi *et al.* (39)
*Apobec*^*−/−*^*;APOB Tg* mouse heps	CD	NR	AAV8-TBG	NA	↓	ND	Musunuru *et al.* (19)
*Apobec*^*−/−*^*;APOB Tg;Ldlr*^*+/−*^ mouse heps	CD	♀	AAV8-TBG	NA	↓	ND	Strong *et al.* (9)
*Apobec*^*−/−*^*;APOB Tg;Ldlr*^*+/−*^ mouse heps	CD	NR	AAV8-TBG	NA	↓	ND	Musunuru *et al.* (19)
WT mice	CD	♀	AAV8-TBG	ND	↓	↑	Strong *et al.* (9)
WT mice	CD	♂	AAV8-TBG	↓	—	ND	Conlon *et al.* (170)
WT mice	CD	♂	AV	↓	ND	ND	Bi *et al.* (39)
WT mice	CD	NR	AAV8	ND	—	ND	Ai *et al.* (40)
WT mice	WD	NR	AV	↑	ND	ND	Kjolby *et al.* (20)
WT mice	HFD	♂	AAV8-TBG	↓	↓	ND	Conlon *et al.* (170)
WT mice	HFD	NR	AAV8	ND	↓	ND	Ai *et al.* (40)
*Apobec*^*−/−*^*;APOB Tg* mice	CD	NR	AAV8-TBG	↓	↓	ND	Musunuru *et al.* (19)
*Apobec*^*−/−*^*;Ldlr*^*−/−*^ mice	CD	NR	AAV8-TBG	↓	ND	ND	Musunuru *et al.* (19)
*Apobec*^*−/−*^*;APOB Tg;Ldlr*^*−/−*^mice	CD	NR	AAV8-TBG	↓	ND	ND	Musunuru *et al.* (19)
*Apobec*^*−/−*^*;APOB Tg;Ldlr*^*+/−*^ mice	CD	NR	AAV8-TBG	↓	ND	ND	Musunuru *et al.* (19)
*Ldlr*^*−/−*^ mice	CD	♀	AAV8-TBG	ND	↓	↑	Strong *et al.* (9)
*Ldlr*^*−/−*^ mice	WD	♂	LV	↑	ND	ND	Lv *et al.* (12)
*Ldlr*^*−/−*^ mice	WD	NR	AV	↑	ND	ND	Kjolby *et al.* (20)
*ob/ob* mice	CD	♂	AV	↓	ND	ND	Bi *et al.* (39)
*ob/ob* mice	WD	NR	AAV8	ND	↓	ND	Ai *et al.* (40)
Downregulation of Sort1							
HeLa T-Rex cells	NA	NA	siRNA	NA	ND	↓	Tveten *et al.* (176)
HepG2 cells	NA	NA	siRNA	NA	ND	↓	Lu *et al.* (159)
HepG2 cells	NA	NA	siRNA	NA	—	ND	Conlon *et al.* (170)
HepG2 cells + FA	NA	NA	siRNA	NA	↑	ND	Conlon *et al.* (170)
A431 cells	NA	NA	siRNA	NA	ND	↓	Lu *et al.* (159)
HUES 1 and 9 HLCs	NA	NA	TALEN KO	NA	↑	ND	Ding *et al.* (177)
McA cells	NA	NA	shRNA	NA	—	ND	Sparks *et al.* (115)
McA cells	NA	NA	siRNA	NA	—	ND	Conlon *et al.* (170)
McA cells - serum-starved	NA	NA	shRNA	NA	↑	ND	Sparks *et al.* (115)
McA cells + FA, Cer, or Tun	NA	NA	siRNA	NA	↑	ND	Conlon *et al.* (170)
hAPOB McA cells	NA	NA	siRNA	NA	↑	ND	Conlon *et al.* (170)
*Sort1*^*−/−*^ mouse heps	WD	NR	Global KO	NA	↓	—	Kjolby *et al.* (20)
*Sort1*^*−/−*^ mouse heps	CD	♂	Global KO	NA	—	ND	Conlon *et al.* (170)
*Sort1*^*−/−*^ mouse heps + FA	CD	♂	Global KO	NA	↑	ND	Conlon *et al.* (170)
*Apobec*^*−/−*^*;APOB Tg;Ldlr*^*+/−*^ mouse heps	CD	NR	siRNA	NA	↑	ND	Musunuru *et al.* (19)
*Sort1*^*−/−*^ mice	CD	♀	Global KO	ND	ND	↓	Strong *et al.* (9)
*Sort1*^*−/−*^ mice	CD	♂	Global KO	—	ND	ND	Goettsch *et al.* (90)
*Sort1*^*−/−*^ mice	CD	♂	Global KO	—	—	ND	Conlon *et al.* (170)
*Sort1*^*−/−*^ mice	CD	NR	Global KO	↑	ND	ND	Musunuru *et al.* (19)
*Sort1*^*−/−*^ mice	CD	NR	Global KO	—	ND	ND	Butkinaree *et al.* (136)
*Sort1*^*−/−*^ mice	CD	♂	Hep KO	—	ND	ND	Chen *et al.* (55)
*Sort1*^*−/−*^ mice	CD	♂	Hep KO	—	—	ND	Conlon *et al.* (170)
*Sort1*^*−/−*^ mice	WD	NR	Global KO	↓	↓	ND	Kjolby *et al.* (20)
*Sort1*^*−/−*^ mice	WD	♂	Hep KO	↓	ND	ND	Chen *et al.* (55)
*Sort1*^*−/−*^ mice	HFD	♂	Global KO	↓	ND	ND	Rabinowich *et al.* (184)
*Sort1*^*−/−*^ mice	HFD	♂	Global KO	↑	↑	ND	Conlon *et al.* (170)
*Sort1*^*−/−*^ mice + Tun	CD	♂	Global KO	ND	↑	ND	Conlon *et al.* (170)
*Apobec*^*−/−*^*;APOB Tg;Sort1*^*−/−*^ mice	CD	♀	Global KO	—	↓	ND	Strong *et al.* (9)
*Apobec*^*−/−*^*;APOB Tg;Sort1*^*−/−*^ mice	WD	♂	Global KO	—	ND	ND	Patel *et al.* (32)
*Apobec*^*−/−*^*;APOB Tg* mice	CD	NR	siRNA	↑	ND	ND	Musunuru *et al.* (19)
*Apobec*^*−/−*^*; APOB Tg; Ldlr*^*−/−*^ mice	CD	NR	siRNA	↑	ND	ND	Musunuru *et al.* (19)
*Apobec*^*−/−*^*;APOB Tg;Ldlr*^*+/−*^ mice	CD	NR	siRNA	↑	ND	ND	Musunuru *et al.* (19)
*Ldlr*^*−/−*^*;Sort1*^*−/−*^ mice	CD	♀	Global KO	↓	ND	—	Strong *et al.* (9)
*Ldlr*^*−/−*^*;Sort1*^*−/−*^ mice	CD	♀	Global KO	—	ND	ND	Hagita *et al.* (178)
*Ldlr*^*−/−*^*;Sort1*^*−/−*^ mice	WD	NR	Global KO	↓	ND	ND	Kjolby *et al.* (20)
*Ldlr*^*−/−*^*;Sort1*^*−/−*^ mice	HF/HC	♀	Global KO	↓	ND	ND	Hagita *et al.* (178)
*Ldlr*^*−/−*^*;Sort1*^*−/−*^ mice	HF/HC	♀	Global KO	—	ND	ND	Goettsch *et al.* (90)
*Ldlr*^*−/−*^*;Sort1*^*−/−*^ mice	HF/HC	♂	Global KO	—	ND	ND	Goettsch *et al.* (90)
*Apoe*^*−/−*^*;Sort1*^*−/−*^ mice	WD	♀	Global KO	—	ND	ND	Mortensen *et al.* (33)
*Apoe*^*−/−*^*;Sort1*^*−/−*^ mice	WD	♂	Global KO	—	ND	ND	Mortensen *et al.* (33)
*L1*^*B6*^*Ldlr*^*−/−*^; *Sort1*^*−/−*^ mice	WD	♂	siRNA	ND	↑	ND	Ai *et al.* (40)
*ob/ob* mice + PBA	CD	NR	siRNA	ND	↑	ND	Ai *et al.* (40)

AAV8-TBG; adeno-associated virus 8 with thyroxine-binding globulin promoter; AV, adenovirus; Cer, ceramide; heps, primary hepatocytes; HLC, hepatocyte-like cell; HUES, human embryonic stem cell line; LV, lentivirus; NA, not applicable; ND, not determined; NR, not reported; PBA, 4-phenyl butyric acid; Tun, tunicamycin; —, no difference.

Summary of published results on the effect of *Sort1* manipulation on lipoprotein metabolism.

Figure 3 diagrams three models that have been proposed to explain the genetic link between sortilin and LDL-C: trafficking VLDL for secretion, trafficking VLDL for degradation, and facilitating LDL clearance. An important review by Dube *et al.* pointed out that while a role for sortilin in VLDL secretion from hepatocytes is plausible, data from human GWAS show a negative association of *SORT1* expression with plasma TC and LDL-C but not with plasma TGs or VLDL (253). Therefore, models that propose control of VLDL secretion as the primary mechanism by which sortilin function influences LDL-C cannot be directly reconciled by the human GWAS.

Intriguingly, a new study by the Rader group published earlier this year indicated that sortilin may also regulate the size of lipoprotein particles secreted from the liver (170). Their main goal was to investigate the effect of sortilin on apoB-100 secretion under basal versus metabolic stress conditions, as discussed further, but observed a disconnect between the effect of sortilin on TG versus total apoB-100 secretion in some of their experiments. For example, overexpression of *Sort1* in CD-fed mice resulted in decreased TG secretion but no difference in the secretion of newly synthesized total apoB-100 in the plasma. To follow up on this surprising observation, they performed sucrose density gradient separation of lipoproteins on plasma pooled from mice of the same genotype taken 2 h after the injection of detergent and ^35^S-Met/Cys. A Western blot of apoB-100 in 11 fractions over a density range of 1.005–1.21 g/ml revealed decreased apoB-100 in the less dense “VLDL” fractions and increased apoB-100 in the higher density “LDL” fractions in the *Sort1*-overexpressing mice. This is consistent with the observed decrease in TG secretion with no difference in apoB-100 secretion because each apoB-100-containing lipoprotein contains one molecule of apoB-100, but the less dense lipoproteins contain more TG. The group found the opposite effect in primary hepatocytes isolated from *Sort1*^*−/−*^ mice: increased TG secretion, no difference in the secretion of total apoB-100, but increased secretion of lower density “VLDL” and decreased secretion of the higher density “LDL.” An indication that sortilin regulates the size of the particles secreted by the liver certainly encourages a more robust study in the future. Interestingly, an earlier article by Ai *et al.* observed increased secretion of apoB-100 without an increase in TG secretion in *Li-Tsc1*^*KO*^ mice, a genetic model of increased hepatic mTORC1 activity by liver-specific knockout of the upstream mTOR inhibitor *Tsc1*, which have 50% less hepatic sortilin mRNA and protein compared with control mice (40). Of the several other mouse models with altered hepatic sortilin expression that they studied (*ob/ob*, diet-induced obesity, *L1*^*B6*^*Ldlr*^*−/−*^), this was the only one that had differential effects on TG and apoB-100 secretion, leading the authors to speculate that the increased secretion of relatively TG-deficient particles was due to the modulation of other pathways involving mTORC1. However, the prospective finding by Conlon *et al.* may support a role of sortilin in this phenomenon.

Recently, sortilin has been indicated as a regulator of Lipoprotein(a) (Lp (a)) secretion; however, this was only demonstrated when sortilin was overexpressed (254). Lp(a) is a variant of LDL that has a second protein, apo(a), covalently attached to apoB-100 (255). A considerable amount of research has been dedicated to determining how Lp(a) is regulated because of the strong relationship between Lp(a) concentration in the plasma and vascular disease (256). Clark *et al.* (254) found that overexpression of sortilin in HepG2 cells that stably express apo(a) increased the secretion of Lp(a) and that this was dependent upon sortilin’s binding to apoB-100. However, knockdown of endogenous sortilin by siRNA had no effect on Lp(a) secretion. The authors propose that this may be due to the modest ∼60% knockdown of sortilin that was achieved by the siRNA. It is also possible that sortilin only has this effect on Lp(a) secretion when expressed at a supraphysiological level or that the experiments were performed in HepG2 cells, which required overexpression of apo(a) because this cell line does not express apo(a) endogenously (257). Further studies are needed to establish the contexts in which sortilin affects Lp(a) secretion.

### Possible explanations for the discrepant results on sortilin’s role in hepatic lipoprotein metabolism

Because of the very strong association between hepatic Sort1 expression level and LDL-C in human GWAS, determining the reason why different functional studies have come to opposing conclusions about the directionality of the effect of sortilin and LDL is of great interest. Because sortilin function is sensitive to many factors, including, but not limited to, its level of overexpression, C-terminal tagging, and the metabolic environment, differences in experimental conditions that influence one or more of these factors are likely to alter the way sortilin traffics apoB-100-containing lipoproteins. There were numerous experimental conditions employed among the studies, and no two studies performed the same measurement under the same experimental conditions (based on the methodological details that were reported). Thorough comparison of the conditions used in each study failed to nominate any one variable to account for the contradictory results between and within research groups.

#### Issues with overexpression and tagging of sortilin

The level of overexpression and the presence of C-terminal tags greatly affect the function of sortilin. In the steady state, sortilin is predominantly localized to the TGN and endosomes, with only a small portion (∼10%) localizing to the cell surface (1, 68, 69, 72, 86). Entry of sortilin into the endolysosomal system from the TGN, recycling back to the TGN, and internalization from the cell surface all require APs (68, 71, 73). High levels of overexpression like that achieved by the constructs used in the majority of the studies can saturate APs like GGA and alter the distribution and localization of sorting receptors like sortilin toward increased levels at the cell surface (252). This has also been shown to occur upon overexpression of VPS10, the yeast homolog of sortilin (65, 99). Strong *et al.* (258) proposed that overexpression of sortilin may also saturate ADAM10, disturbing the balance of full-length sortilin and soluble sortilin abundances in the cell, contributing to the inconsistent results. The soluble form of sortilin, generated by ADAM10 at the plasma membrane and also to some extent in the secretory pathway, binds ligands with similar affinity to full-length sortilin (110, 112, 120). Since soluble sortilin retains the ability to bind ligand, it has been postulated that it could function as a “decoy receptor” in the secretory pathway and extracellularly. Regulating sortilin cleavage by ADAM10 can be a way for the cell to control the amount of ligand available to bind to full-length sortilin or other receptors that share the same ligand. C-terminal tagging of sortilin can also alter its distribution and localization. GGA proteins are critical to the proper trafficking of sortilin in endosomes (68, 69, 93, 94), and placement of a tag at the C-terminus of sortilin interferes with the binding to GGA (94).

#### Differences in the tissue specificity of the methods used to manipulate *Sort1* levels

Another factor that could contribute to the discrepant findings is the level by which different methods of overexpression, knockdown, and knockout of *Sort1* occurred in nonhepatic tissues in experiments conducted by various research teams. Sortilin has lipid-related functions in cells other than hepatocytes, such as its role in mediating the internalization and degradation of lipoprotein lipase (LPL) (11), intestinal cholesterol absorption (178), intestinal fatty acid absorption via its function as a neurotensin receptor (183), as discussed in later sections.

A recent article by Chen *et al.* was the first to publish results from a tissue-specific *Sort1*^*−/−*^ mouse. The study found that hepatocyte-specific knockout of sortilin (by breeding *Sort1*^*flox/flox*^ mice to mice expressing Cre driven by the hepatocyte-specific albumin promoter) results in a 22% decrease in plasma TC in mice fed a WD for 12 weeks. This suggests that the results showing a lowering of plasma TC or LDL-C upon knockdown or knockout of *Sort1* obtained by Kjolby *et al.*, Hagita *et al.*, Goettsch *et al.*, and Strong *et al.* are due to altering the abundance of sortilin in hepatocytes. However, this is the opposite directionality of the prediction from human GWAS, namely that decreased hepatic *Sort1* levels would result in increased LDL-C (14, 19, 36).

#### Use of different *Sort1*^*−/−*^ mouse models

Three different global *Sort1*^*−/−*^ mice were used between the studies: one that was generated by replacing a segment between exon 2 and intron 3 of the *Sort1* gene with a neomycin-resistance cassette (9, 19, 32, 136)), another that was made by replacing of a fragment from exon 14 and the subsequent intron with Neo (20, 33, 119, 184), and a third that was made by targeted deletion of a fragment of exon 14 (90, 178). The latter two models likely express a truncated protein consisting of part of the luminal domain that may fold and be able to bind ligand.

#### Differences in metabolic context—genetic background, age, sex, and diet of mice, and cell culture conditions

Since the expression and trafficking of sortilin is affected by levels of insulin resistance (40, 57, 58), ER stress (40, 58), saturated fatty acids (39, 56, 61), glucose (50), oxidized LDL (12), and inflammatory cytokines (41, 56), it is likely that metabolic context influences the directionality by which sortilin affects LDL-C. Many of the differences in experimental conditions used in the studies can greatly influence these parameters, including the genetic background of the mice being studied (e.g., WT, *Apobec*^*−/−*^, APOB Tg, *Ldlr*^*−/−*^, *Apoe*^*−/−*^, *ob/ob*), the diets used (e.g., CD, WD, high-fat/high-cholesterol (HF/HC) diet, high-fat diet (HFD)), the age at which the mice were started on experimental diet, the length of diet feeding, the sex of the mice, and the culture conditions in studies using cell lines. Some studies used only female mice, whereas others used only males, with very few studying both. In many cases, the sex of the mice used was not reported. C57BL/6 mice were used in all the studies, but report of the specific substrain (e.g., the phenotypically different and widely studied J and N lines) was often lacking. In a case where these details were provided, mice of a mixed C57BL/6J and C57BL/6N background were used (55).

Studies by the Sparks group using the McArdle RH7777 (McA) rat hepatocyte cell line have clearly demonstrated how cell culture conditions that affect insulin sensitivity alter the role of sortilin in VLDL secretion. Insulin suppresses the secretion of VLDL and apoB-100 by favoring the presecretory degradation of apoB-100 (259, 260, 261, 262). Under serum-enriched conditions, McA cells are insulin resistant, and under serum-starved conditions, they are insulin sensitive. Under baseline conditions in the insulin-sensitive state, sortilin facilitates VLDL secretion. Upon addition of insulin in this state, sortilin shifts to facilitating the insulin-dependent presecretory degradation of VLDL, potentially through a mechanism involving binding of the insulin-signaling molecule phosphatidylinositol (3,4,5)-triphosphate to the luminal domain of sortilin (155). However, in the insulin-resistant state of the cells (serum-enriched conditions), VLDL secretion and degradation is regulated independently of sortilin (114, 115).

There were three groups that conducted experiments on both chow- and either WD- or HFD-fed mice in the same study: Ai *et al.* (40), Chen *et al.* (55), Conlon *et al.* (170). All three found that WD or HFD feeding was required for manipulation of *Sort1* expression to have an effect on apoB-100 secretion and/or plasma TC. Both Conlon *et al.* and Ai *et al.* presented data showing that this unmasking of a role for sortilin in apoB-100 secretion under HFD-fed conditions was due to the increased level of ER stress in the livers of the diet-fed mice (40, 170).

Many reviews have endorsed the idea that sortilin acts through distinct mechanisms that can raise and lower LDL-C (trafficking VLDL for secretion, trafficking VLDL for degradation, and facilitating LDL clearance) and that the balance of these actions in a specific metabolic environment determines its contribution to circulating levels of LDL-C (175, 253, 258, 263, 264, 265).

#### *SORT1* may not be the only important gene

Even though *SORT1* has been the primary functional candidate gene at the 1p13.3 locus for the association with LDL-C in GWAS, its neighboring genes, *CELSR2* and *PSRC1*, are also regulated by the causal SNPs, making it possible that multiple genes at the locus act together to regulate LDL levels (14, 19, 36, 266). In fact, a study published earlier this year that analyzed published GWAS and quantitative trait locus studies using Mendelian randomization methods found that higher expression levels of *SORT1*, *PSRC1*, and *CELSR2* in liver were all individually significantly associated with lower LDL-C and coronary artery disease (CAD) risk (267). Similarly, in another article published this year, analysis of allelic ratios built from tissue-specific RNA sequencing data available through the human Genotype-Tissue Expression Project (GTEx) indicated that the multiple SNPs at the 1p13.3 locus likely regulate more than one gene to account for the predicted disease risk (268).

The Musunuru group has investigated developing alternative models to those that have been used to study the role of sortilin in hepatic lipoprotein metabolism and began by returning to the original human GWAS observation. Unfortunately, the minor allele of rs12740374 does not create a C/EBPα binding site in mice as it does in humans. The group explored the potential usefulness of three different model systems: *1*) a collection of primary human hepatocytes with varied rs12740374 genotypes, *2*) a population cohort of induced pluripotent stem cell-derived hepatocyte-like cells, and *3*) a mouse model where the human 1p13.3 locus containing the rs12740374 minor allele has been incorporated into the genome via bacterial artificial chromosome transgenesis (269). Initial experiments indicated that primary human hepatocytes and bacterial artificial chromosome transgenic mice could be better alternatives to the current model systems for studying how the common human 1p13.3 SNP affects lipoprotein metabolism.

### Potential roles for sortilin in cholesterol metabolism outside of hepatic lipoprotein trafficking

Although much of the studies investigating the role of sortilin in cholesterol metabolism revolves around its trafficking of lipoproteins in the hepatocyte, its involvement in intestinal cholesterol and fatty acid absorption and bile acid synthesis may also affect circulating cholesterol levels.

#### Intestinal cholesterol and fatty acid absorption

There is evidence that sortilin plays a role in intestinal cholesterol absorption, which contributes to plasma cholesterol levels. Female *Sort1*^*−/−*^*Ldlr*^*−/−*^ mice fed an HF/HC diet have elevated fecal TC, suggesting impaired intestinal cholesterol absorption (178). Sortilin deficiency decreases intestinal mRNA levels of Niemann-Pick C1-like intracellular cholesterol transporter 1 (*Npc1l1*), an intestinal cholesterol transporter, liver X receptor (LXR), a key transcriptional lipid metabolism regulator, and of several of their regulators. It is unknown how sortilin deficiency affects the mRNA levels of these genes, but because sortilin is a trafficking receptor, it is likely that this effect on transcription is indirect. In addition, it is difficult to determine whether these changes are a cause of the reduced fecal TC in the mice or a result of it. Even though it was not evaluated in this study, the possibility that sortilin directly participates in intestinal cholesterol absorption by trafficking proteins involved in this process cannot be ruled out. In addition, one of sortilin’s most well-studied ligands, neurotensin, has been shown to play a role in HFD-induced obesity by increasing fat absorption in the intestine (183). It is possible that sortilin is involved in this process by regulating neurotensin levels.

#### Bile acid synthesis

Sortilin may regulate cholesterol metabolism by playing a role in bile acid synthesis. Bile acids are synthesized from free cholesterol, stored in the gall bladder, and released into the intestine to facilitate digestion and absorption of lipids in the small intestine as well as to regulate cholesterol homeostasis (270, 271). Cholestasis occurs when flow of bile out of the liver is impaired as a result of decreased secretion from the hepatocytes or obstruction of bile flow (272, 273) and can lead to liver inflammation, steatohepatitis, fibrosis, and cirrhosis (274, 275, 276). In the initial stages of cholestatic injury, a ductular reaction occurs, characterized by the proliferation of reactive bile ducts, myofibroblast activation, and an influx of inflammatory cells (274, 277, 278).

A study by Li *et al.* discovered that *Sort1*^*−/−*^ mice are protected from high cholesterol/cholate diet-induced liver injury because of increased levels of carboxylesterase 1 (CES1) in the liver, promoting bile acid synthesis (13). Sortilin binds and traffics CES1 to the lysosome for degradation. The livers of *Sort1*^*−/−*^ mice have increased CES1 protein, and decreased accumulation of free cholesterol and knockdown of hepatic CES1 abolished the protective effect of *Sort1*^*−/−*^ on liver injury. In addition, the high cholesterol/cholate diet-fed *Sort1*^*−/−*^ mice also had increased hepatic cytochrome P450 family 1 subfamily A member 1 (*Cyp7a1*) expression, the rate limiting enzyme in the conversion of cholesterol to bile acids, likely contributing to the increased bile acid synthesis in these mice. Kjolby *et al.* (20) also observed this increase in *Cyp7a1* expression in the livers of *Sort1*^*−/−*^ mice. Although the *Sort1*^*−/−*^ mice have increased bile acid synthesis, the rate of secretion of bile acids from the liver into the gallbladder is normal, resulting in an accumulation of bile acids in the liver. Interestingly, despite this buildup of bile acids in the liver, the mice are protected from liver injury. This may be explained by the findings in a follow-up article from the group, and of another study by Hubel *et al.*, that discovered that *Sort1*^*−/−*^ mice are also protected from liver injury after bile duct ligation, a model of obstructive cholestasis, and have a reduced ductular reaction 3 days after bile duct ligation through a mechanism that involves the role of sortilin in the secretion of the proinflammatory cytokine interleukin 6 (IL-6) (279, 280). As discussed in a later section, sortilin is involved in the secretion of IL-6 and other proinflammatory cytokines from immune cells (33, 48).

A study by Chen *et al.* (55) also assessed bile acid pools in *Sort1*^*−/−*^ mice. In contrast to Li *et al.*, they found that hepatocyte-specific *Sort1*^*−/−*^ mice had normal gallbladder, intestine, and liver bile acid contents and pool sizes, as well as normal hepatic expression of *Cyp7a1*. However, the mice in the study by Chen *et al.* were fed a WD for 12 weeks, whereas the mice in the study by Li *et al.* were fed a high cholesterol/high cholate diet. It is therefore likely that the high cholesterol/high cholate diet is required to reveal the effects of sortilin on bile acid metabolism.

### Involvement of sortilin in CAD independent of its effect on circulating LDL-C levels

In addition to the SNPs at the 1p13.3 *CELSR2-PSRC1-SORT1* locus being strongly associated with circulating LDL-C levels, this locus is also associated with several contributors of CAD, including atherosclerosis and arterial calcification (17, 18, 229, 244, 246, 281, 282, 283, 284, 285, 286, 287, 288, 289, 290, 291, 292, 293). While LDL-C is a major risk factor for atherosclerosis, studies have found that sortilin may play a role in the development of atherosclerosis independent of its effect on circulating LDL-C levels.

#### Macrophage cytokine secretion, cholesterol efflux, and foam cell formation

Independent of the effect of sortilin manipulation on plasma TC and LDL-C levels, knockout of *Sort1* in mice reduces the development of atherosclerotic lesions (32, 33). Transfer of bone marrow from *Sort*^*−/−*^ mice into irradiated *Apoe*^*−/−*^ (33) or *Ldlr*^*−/−*^ (32) atherosclerotic mice reduced their atherosclerosis, implying that this effect is mediated by sortilin’s function in macrophages. The mechanism behind this effect, however, is debated. Mortensen *et al.* (33) discovered that sortilin promotes both the secretion and the internalization of the proinflammatory cytokines IL-6 and IFN-γ in macrophages, molecules that mediate atherosclerotic plaque formation. In this study, *Sort1* deficiency did not influence macrophage recruitment or foam cell formation. Patel *et al.* (32) also found no effect of *Sort1* deficiency on macrophage recruitment. However, in contrast to Mortensen *et al.*, they showed no effect of sortilin deficiency on cytokine secretion. In addition, they found that macrophages from *Sort1*^*−/−*^ mice had decreased uptake of LDL and reduced foam cell formation, and overexpression of *Sort1* had the opposite effect. The reason for the discrepancy is not known; however, mice of different genetic backgrounds were used in the studies. The finding by Patel *et al.* that *Sort1* deficiency results in increased LDL uptake and foam cell formation in the absence of the LDLR could potentially explain the ability of LDL to promote macrophage foam cell formation independently of the LDLR. They also found that LDL uptake increases sortilin levels in macrophages, suggesting a feed-forward loop.

Lv *et al.* (12) discovered that sortilin also promotes aortic atherosclerosis by inhibiting cholesterol efflux from macrophages by targeting the ATP-binding cassette subfamily A member 1 (ABCA1) transporter for lysosomal degradation. ABCA1 is a transmembrane protein that transports cellular cholesterol from cells to lipid-poor apoA-I-containing lipoproteins. Overexpression of *Sort1* with lentivirus in the THP-1 monocyte-like cell line and mouse peritoneal macrophage-derived foam cells decreased apoA-I-mediated cholesterol efflux by suppressing the expression of the ABCA1 transporter. Treatment with shRNA against *Sort1* had the opposite result. Lentiviral overexpression of *Sort1* in *Ldlr*^*−/−*^ mice resulted in decreased plasma HDL-C, increased plasma LDL-C, and an increase in atherosclerotic lesions and lipid deposition.

#### Smooth muscle cell apoptosis

The function of sortilin as a coreceptor for proNGF-mediated p75NTR cell death signaling may play a role in the progression of atherosclerosis in addition to its well-known role in neuronal diseases (27, 121). Campagnolo *et al.* (179) revealed that sortilin is upregulated in human fibroatheromatous plaques compared with normal young vessels and in cultured rat aortic intimal cells compared with normal media smooth muscle cells (SMCs). The study found that knockdown of *Sort1* in rat intimal cells protects them from proNGF-induced apoptosis suggesting that sortilin represents an important regulator of proNGF-induced SMC apoptosis and arterial remodeling and through this action may contribute to the progression of atherosclerosis.

#### Vascular calcification

Vascular calcification is associated with atherosclerotic plaques and can cause the plaques to rupture, leading to myocardial infarction. There are two types of vascular calcification: microcalcification and macrocalcification (294, 295). The two types are differentially regulated and have different clinical risks. Extracellular vesicles (EVs) participate in the formation of microcalcifications (296), which usually form vulnerable plaques and are implicated in atherosclerotic plaque rupture and acute cardiovascular events (297). These EVs, also called matrix vesicles, are secreted by vascular smooth muscle cells (VSMCs) and macrophages (296, 298, 299) and aggregate to serve as initial sites for mineral formation. Macrocalcifications have increased plaque stability, and long-term macrocalcifications can lead to decreased vascular integrity and subsequent heart failure (300).

*Sort1* is upregulated in human mesenchymal stem cells during osteoblastic differentiation (180, 181), a process that VSMCs undergo as they calcify. Overexpression of *Sort1* in human mesenchymal stem cells results in the acceleration of mineralization during osteogenic differentiation (180). In addition, *Sort1* expression is upregulated in calcified arterial tissues in mouse models of arterial calcification, and treatment of VSMCs with shRNA against *Sort1* inhibits β-glycerophosphate-induced mineralization (51).

Using both cell lines and mouse models, Goettsch *et al.* (90) discovered that sortilin promotes vascular microcalcification by trafficking tissue-nonspecific alkaline phosphatase into EVs in SMCs. Extracellular pyrophosphate is one of the main inhibitors of calcification, and when it is hydrolyzed to phosphate, mainly by tissue-nonspecific alkaline phosphatase (TNAP) secreted in EVs from SMCs, it promotes calcification (301, 302).

A study by Sun *et al.* found an additional mechanism by which sortilin is involved in vascular calcification by affecting EVs. The receptors galectin-3 and a receptor for advanced glycation end products differentially regulate the formation of microcalcification and macrocalcification upon binding of advanced glycation end products, including Nε-carboxymethyl-lysine (303). Sun *et al.* (303) found that this regulation of the different vascular calcification types was partially mediated through the receptors’ regulation of sortilin expression. In VSMCs, receptor for advanced glycation end product signaling decreased sortilin expression and mediated the formation of microcalcification, whereas galectin-3 signaling increased sortilin expression and induced macrocalcification. This effect of sortilin promoting macrocalcification disagrees with that of Goettsch *et al.*, who found that sortilin promotes microcalcification. However, Sun *et al.* also found that sortilin expression accelerated the accumulation of matrix vesicles, which are involved in microcalcification. Therefore, both studies agree that sortilin promotes vascular calcification, but the exact mechanisms are not fully elucidated. It is possible that sortilin promotes macrocalcification in certain biological contexts and microcalcification in others.

### Summary of the role of sortilin in LDL-C metabolism and CAD

A potential role for sortilin in cholesterol metabolism and CAD was originally suggested when human GWAS studies discovered that SNPs regulating *Sort1* expression in the liver are strongly associated with circulating LDL-C levels and several aspects of CAD, including atherosclerosis and vascular calcification. Follow-up studies by several groups have found that sortilin influences circulating LDL-C levels by trafficking apoB-100-containing lipoproteins in the hepatocyte but do not agree on the directionality of the effect. Follow-up studies revealed that sortilin is involved in several aspects of atherosclerosis progression, including the regulation of circulating cholesterol levels by affecting trafficking of apoB-100-containing lipoproteins in the liver, intestinal cholesterol absorption, and bile acid synthesis, as well as through its involvement in macrophage cytokine secretion, macrophage cholesterol efflux, and foam cell formation.

### Role of sortilin in glucose homeostasis, insulin resistance, and obesity

The role of sortilin in cardiovascular risk and metabolic disease goes far beyond the GWAS finding of its association with LDL-C. Although not corroborated by human genetic data yet, sortilin has been shown to play a role in the control of glucose homeostasis, insulin resistance, and obesity.

#### GLUT4 trafficking and insulin-mediated glucose uptake

Several studies using adipocyte and myocyte cell lines have demonstrated that sortilin is a major protein component of glucose transporter 4 (GLUT4) storage vesicles (GSVs) (85, 147, 151) and plays a role in their biogenesis (83, 152). It is likely that the mechanism by which sortilin is involved in GSV biogenesis is through the binding of GLUT4 by its luminal domain and the APs AP1 and GGA by its cytoplasmic domain to traffic GLUT4 into vesicles (148, 304, 305, 306, 307). Recently, one group has discovered that the abundance of an alternatively spliced sortilin variant that includes the alternative exon 17b (Sort^17b^) increases with insulin resistance in mouse 3T3L1 adipocytes, the implications of which are still to be determined (308). In C2C12 myotubes, saturated fatty acids induce downregulation of sortilin in a PKC-dependent manner. This downregulation of sortilin was shown to impair GLUT4 trafficking in these cells, providing a potential mechanism of PKC-dependent insulin resistance (61).

More recently, it was discovered that sortilin also plays a significant role in the retrograde transport of GLUT4. Under normal conditions, when insulin levels begin to decrease, GLUT4 is internalized and recycled from endosomes back to the TGN to be resorted into GSVs. However, under insulin-resistant conditions where there is long-term insulin stimulation, instead of being recycled, GLUT4 is transported to the lysosome for degradation (309, 310, 311, 312). Sortilin and retromer binding to its cytoplasmic tail are important for retrieval of GLUT4 from endosomes (150, 313). Long-term insulin stimulation induces dissociation of retromer components from sortilin and endosomal membranes of 3T3-L1 adipocytes, inhibiting recycling of GLUT4 (150, 313). Consistent with this, in the absence of *Sort1* in 3T3-L1 adipocytes, GLUT4 is degraded (83).

The in vitro data on the importance of sortilin in GSV biogenesis would predict that *Sort1*^*−/−*^ mice would be severely insulin resistant in the context of insulin-stimulated glucose uptake into adipose tissue and skeletal muscle and likely diabetic. Paradoxically, in vivo data suggest that *Sort1*^*−/−*^ mice have either normal or *increased* insulin sensitivity, depending on the study, as assessed by hyperinsulinemic-euglycemic clamps, glucose tolerance tests, and insulin tolerance tests (59, 184). In addition, the level of GLUT4 protein in WAT and skeletal muscle from WT and *Sort1*^*−/−*^ mice is similar (59), inconsistent with the finding that GLUT4 levels are decreased upon *Sort1* silencing in 3T3-L1 adipocytes (83).

The reasons behind the drastic differences in results between in vitro and in vivo experiments on the role of sortilin in insulin-stimulated glucose disposal are unclear. Since the *Sort1*^*−/−*^ models used were whole-body chronic knockouts, it is possible that the mice develop a compensatory mechanism (or overcompensate) for the lack of sortilin. It is also possible that the cell lines used in the in vitro studies do not possess the exact same regulatory components of insulin-stimulated glucose disposal as adipocytes and myocytes in vivo do. To date, there are published studies neither on adipose- or muscle-specific *Sort1*^*−/−*^ mice nor inducible *Sort1*^*−/−*^ mice.

#### Obesity and insulin resistance

Studies in mouse models and adipocyte cell lines have provided evidence that sortilin plays a role in the development of adiposity, albeit with some controversial results. Just as with the discrepant results between groups regarding sortilin’s role in lipoprotein metabolism, the use of different *Sort1*^*−/−*^ mouse models, background, sexes, and feeding different diets at different ages and for various lengths of time may be the culprit. Hagita *et al.* fed female and male *Sort1*^*−/−*^ mice, generated via targeted deletion of a region of exon 14, on a *Ldlr*^*−/−*^ background either chow diet or an HF/HC diet for 15 weeks starting at 10 weeks of age (178). The female *Sort1*^*−/−*^ mice had significantly lower body weight on both the chow and HF/HC diet, starting 3 weeks after the start of diet feeding, with no difference in food intake. At sacrifice, these mice had lower WAT mass with smaller adipocytes. The male *Sort1*^*−/−*^ mice gained a normal amount of weight on either chow or HF/HC diet over the entire course of the study. A study by Li *et al.* not only also reported normal weight gain in male *Sort1*^*−/−*^ mice fed chow diet on a WT background but also observed no body weight phenotype when these mice were fed a WD for 8 weeks starting at 10 weeks of age (59). Another study by the same group found normal body weight gain in female *Sort1*^*−/−*^ mice on a WT background fed a high cholesterol/cholate diet for 6 weeks (13). This group used a different *Sort1*^*−/−*^ mouse than that of Hagita *et al.*, generated using gene trapping to insert a stop codon into intron 2. Studies by Rabinowich *et al.* and Conlon *et al.* showed male *Sort1*^*−/−*^ mice on a WT background fed HFD for 10–12 weeks but reported opposing effects on body weight. Rabinowich *et al.* (184) found decreased body weight, visceral fat, and liver fat in the *Sort*^*−/−*^ mice, whereas Conlon *et al.* (170) reported a trend for increased body weight, although not statistically significant. The only obvious difference between the reported methods in these two studies was the use of different *Sort1*^*−/−*^ mouse models. Rabinowich *et al.* studied a *Sort1*^*−/−*^ mouse generated by introducing a Neo cassette into exon 14 and the following intron, and Conlon *et al.* studied a *Sort1*^*−/−*^ mouse made by replacing a segment between exon 2 and intron 3 with a Neo cassette. One hypothesis may be that the targeting of an early versus late exon is the cause for the differences, but comparison of the *Sort1*^*−/−*^ mouse models and phenotypes between the four groups (Hagita *et al.*, Li *et al.*, Rabinowich *et al.*, and Conlon *et al.*) does not support this.

Neurotensin knockout (*Nts*^*−/−*^) mice share many of the phenotypes described for *Sort1*^*−/−*^ mice in the studies by Hagita *et al.* and Rabinowich *et al.*, including protection from obesity, hepatic steatosis, and metabolic disorders (183). It is possible that the function of sortilin as a neurotensin receptor contributes to these phenotypes in the *Sort1*^*−/−*^ mice. In the intestine, neurotensin promotes fatty acid absorption, and this effect has been shown to occur through attenuation of the activation of 5′ adenosine monophosphate-activated protein kinase (AMPK) through a mechanism involving a complex of neurotensin receptor 1 (NTR1) and sortilin (183).

Another mechanism by which sortilin may influence adiposity is through adipocyte differentiation. Sortilin expression in human preadipocytes dramatically increases upon differentiation into adipocytes (182), and overexpression of *Sort1* in the adipocyte precursor model cell line 3T3-L1 was sufficient to inhibit adipocyte differentiation upon induction, as assessed by lipid accumulation (95). Baltes *et al.* discovered that sortilin binds and colocalizes with delta-like noncanonical Notch ligand 1 (DLK1), a receptor known to negatively regulate adipocyte differentiation, and that DLK1 expression is altered in *Sort1*-overexpressing 3T3-L1 cells. Although they did not have any experiments to directly test their hypothesis, they propose that sortilin inhibits adipogenesis in 3T3-L1 cells by affecting the trafficking of DLK1.

Interestingly, work by Chen *et al.* (55) studying male hepatocyte-specific *Sort1*^*−/−*^ mice, where albumin-Cre recombination resulted in deletion of exons 2 and 3 and a subsequent frameshift, found that *Sort1* knockout resulted in slower weight gain upon WD feeding, with significant reductions in weight beginning 9 weeks after the start of the diet. This indicates a potential mechanism by which sortilin function in the liver affects WAT size that merits further investigation.

### Summary of the role of sortilin in cardiovascular and metabolic disease

**Fig. 4:**
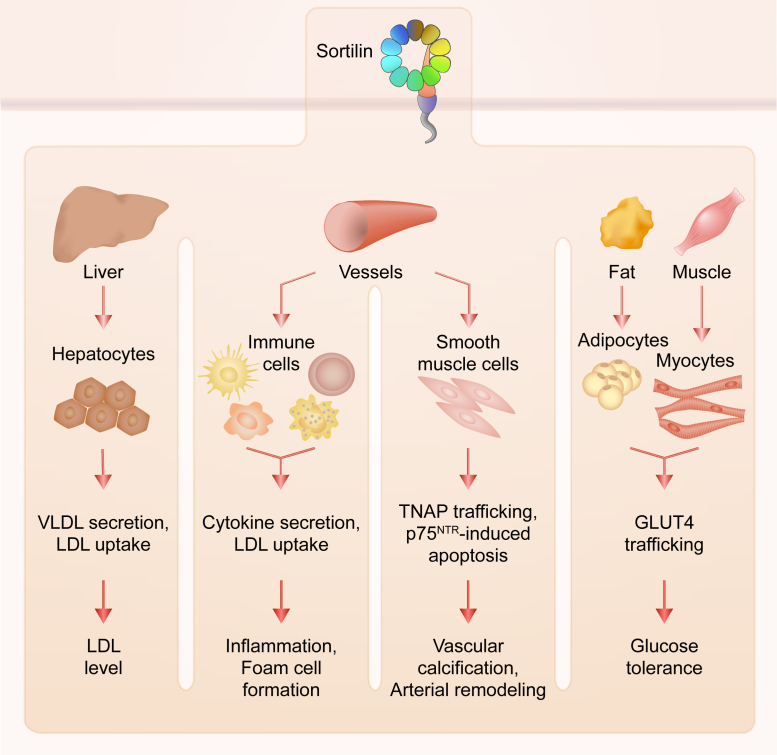
Sortilin contributes to many aspects of cardiovascular risk. Human GWAS, physiological studies in mice, and biochemical studies in cells have revealed a role for sortilin in several aspects of cardiovascular risk. Sortilin regulates LDL-C level by trafficking apoB-100-containing lipoproteins in hepatocytes, inflammation, and foam cell formation by regulating cytokine secretion and lipid uptake in macrophages, vascular calcification, and arterial remodeling by trafficking TNAP and p75NTR-induced apoptosis in smooth muscle cells, and glucose tolerance by trafficking GLUT4 storage vesicles in fat and muscle cells. Diagram adapted from Goettsch *et al.* (314).

The function of sortilin in various tissues and cell types influences the risk for CVD (Fig. 4). Human GWAS first revealed an unusually strong association between regulatory SNPs near the *SORT1* locus, *SORT1* expression, and LDL-C, a major risk factor for atherosclerosis. Studies in animal models have established a role for sortilin in trafficking apoB-100-containing lipoproteins in hepatocytes but have generated conflicting results regarding the directionality of the trafficking and the effect on LDL-C. The SORT1 locus is independently associated with several other contributors of CAD, including the progression of atherosclerosis through its role in macrophage cytokine secretion, cholesterol efflux, and foam cell formation, and affects atherosclerotic plaque development by regulating SMC apoptosis and vascular calcification. Although not corroborated by human genetic data yet, sortilin has been shown to play a role in glucose homeostasis, insulin resistance, and obesity, likely through its trafficking of GLUT4 storage vesicles.

### Sortilin as a potential drug target

The role of sortilin in disease makes it a potential therapeutic drug target, but it is a complicated target because of its involvement in numerous biological pathways in a multitude of tissues and cell types. Inhibiting sortilin would affect many pathways, some of which may contribute to disease, and others that may protect from disease. There are several approaches one could take to target sortilin. A number of groups have investigated blocking ligand binding to sortilin with small-molecule inhibitors. Neurotensin is the only ligand that has been crystallized with sortilin, revealing its binding site within a small pocket in the tunnel of the β-propeller structure of sortilin’s VPS10 domain (25). However, there is evidence for multiple distinct binding sites on sortilin, as demonstrated by the failure of neurotensin to block binding of most ligands. This may be beneficial to drug design, as synthesizing a molecule designed to inhibit binding of some ligands would likely not inhibit binding of all ligands. Mapping binding sites for each of sortilin’s ligands would be a prerequisite to determine the ligands whose binding would be inhibited by a specific small molecule and to anticipate potential side effects.

Small molecules that bind to the neurotensin-binding site of sortilin have been developed. As mentioned previously, the small pocket in the VPS10 domain of sortilin where neurotensin binds has been described to have two binding sites, one that binds to the N-terminal half of neurotensin, and the other that binds to the C-terminal half of neurotensin (25, 132). The C-terminal half of neurotensin contributes the most to its binding, as the N-terminal half does not bind sortilin on its own but seems to enhance the binding affinity of full-length neurotensin (25, 132). AF40431 was the first small-molecule ligand reported to bind sortilin (315). It binds in the same location as the C-terminal end of neurotensin and inhibits neurotensin binding and likely other ligands that bind to the same site. Unfortunately, AF40431 has very low solubility and membrane permeability. This led to the development of its optimized successor, AF38469, which binds in the same location as the C-terminal end of neurotensin, but is orally bioavailable, has increased membrane permeability, has a similar affinity for sortilin as that of neurotensin, and seems to be selective for sortilin, as demonstrated by its inability to bind NTR1 and a panel of other proteins known to bind similar molecules (316).

Several groups have used AF38469 in biological experiments. As previously discussed, Rhost *et al.* discovered that sortilin-mediated endocytosis of progranulin is required for progranulin to induce metastasis of breast cancer cells. Delivery of AF38469 into MDA-MB-231 human breast cancer cell-xenografted mice via their drinking water during tumor growth completely inhibited progranulin-induced metastasis of the breast cancer cells into the lung (212). Yang *et al.* demonstrated that sortilin promotes glioblastoma invasion, and treatment with AF38469 attenuated the migration of glioblastoma cell lines and decreased glioblastoma tumor growth and invasive capacity in mice (217). In addition, the survival time of the mice treated with AF38469 was significantly longer than that of mice in the control group (28.5 vs. 18.5 days). Therefore, inhibition of ligand binding to sortilin with AF38469 may be a potential therapy for several cancers.

The effect of AF38469 on aspects of CVD has also been investigated. Feeding of WT mice with AF38469-supplemented WD did not affect body weight gain but did result in significantly decreased plasma TC, because of a decrease in hepatic VLDL secretion (55). Importantly, AF38469 treatment did not affect plasma aspartate aminotransferase and alanine aminotransferase levels, suggesting that it is not hepatotoxic. The ability of the inhibitor to reproduce the decreased TC but not the reduced weight gain that was observed in the *Sort1*^*−/−*^ mice may suggest that the ligands responsible for the effect on weight gain bind to a different site on sortilin than AF38469 (and neurotensin). AF38469 did not affect fasting plasma glucose, insulin, or free fatty acid concentration or glucose tolerance during a glucose tolerance test in the WD-fed mice. Therefore, treatment with AF38469 to inhibit ligand binding to sortilin may be useful as a therapy for hypercholesterolemia but not for insulin resistance.

Sparks *et al.* (115, 155, 168) developed another small molecule that binds to the C-terminal neurotensin binding site of sortilin, called cpd541, as well as the first small molecule that binds the N-terminal neurotensin binding site, cpd984. Similar to AF38469, cpd541 inhibits neurotensin binding (168). However, remarkably, cpd984 *enhances* neurotensin binding to sortilin (115). Interestingly, cpd984 also enhances the binding of other ligands to sortilin through the C-terminal neurotensin binding site, including phosphatidylinositol (3,4,5)-triphosphate (155), and likely apoB-100 (115). As discussed previously, the group developed various insulin-sensitive McA hepatoma cell lines in which sortilin levels were differentially reduced with siRNA. They found that the amount of VLDL secreted from the cells was positively correlated with sortilin level, indicating that sortilin facilitates VLDL secretion in insulin-sensitive McA cells. Treatment with cpd984 enhanced VLDL secretion in all the cell lines, but in proportion to the amount of expressed sortilin, suggesting that sortilin remains rate limiting. These data suggest that cpd984 increases the interaction of VLDL with sortilin and that VLDL binds to the same site as the C terminus of neurotensin. In contrast, treatment with cpd541 reduced VLDL secretion, consistent with the C-terminal neurotensin binding site being the primary region of VLDL binding to sortilin. These studies suggest that small-molecule ligands of sortilin can be designed not only to inhibit the function of sortilin but also to enhance its activity and could be used as a therapeutic for diseases in which sortilin activity is beneficial, as in some types of cancers.

Altering the effects of sortilin could also be achieved by targeting a ligand, instead of the receptor. For example, work by Andersen *et al.* (315) discovered a region on the surface of sortilin that is essential for proneurotrophin binding but not for the binding of neurotensin. The group demonstrated that a synthesized peptide containing the proneurotrophin-binding sequence in sortilin effectively blocked proneurotrophin binding and inhibited pro-NGF-induced cell death in the schwannoma cell line RN22, presumably by binding to and blocking the site on the proneurotrophin that would normally bind full-length sortilin. The peptide did not affect sortilin hetero-oligomerization with p75NTR, and it would not affect the binding of proneurotrophins to the p75NTR because sortilin and p75NTR bind to different regions of proneurotrophins. Unfortunately, the authors point out that the peptide is unlikely to be suitable for therapeutic use because of its modest affinity and therefore high concentration required to inhibit proneurotrophin binding. However, optimization of this peptide to improve its binding affinity to proneurotrophins, as well as enhance solubility, stability, and efficacy could lead to a drug that would inhibit proneurotrophin-induced apoptosis while leaving the trophic action of mature neurotrophins unaffected.

Future therapeutic strategies to target sortilin would need to be as tissue- and pathway specific as possible, as targeting sortilin’s function could be beneficial in one tissue or pathway but detrimental in another, and this may also depend on the disease state. Nanotechnology-based drug targeting and antisense oligonucleotide conjugation to tissue-specific ligands may prove useful to target molecules to specific tissues or cell types.

## Conclusions

The GWAS association of SNPs near *SORT1* remains among the strongest associations with LDL-C and coronary heart disease. However, a strong genetic association does not always shed light on an easily decipherable biological mechanism. This is similar to the very strong association and effect size of the *APOE4* allele with the risk of Alzheimer’s disease. Despite more than three decades of intensive research, there is still no clear consensus on the mechanism behind this strong association.

The length and complexity of this review reflects the complexity of sortilin itself. Sortilin’s complexity is due in part to its large number of ligands. These ligands have known roles in multiple biological processes, including lipid metabolism, cell growth, immune function, neurobiology, and glucose homeostasis. Many studies on sortilin have reached divergent conclusions. Analysis of these studies is difficult because the experimental conditions were often not identical. The in vivo experiments employed three different knockout mouse models. Some of the studies were done in mixed strain backgrounds and others in a pure background, whereas others were in undefined backgrounds. The cell line experiments often involved overexpression of sortilin clones. Some of these clones were tagged at the C terminus, and such tags have been shown to interfere with proper subcellular trafficking of sortilin because of interference with the binding of GGA2. Sortilin is normally expressed at a very low level in hepatocytes. Overexpression of the founding member of sortilin’s gene family, yeast VPS10, has been shown to titrate out its binding partners and cause its mislocalization. Thus, overexpression in liver cell lines may have caused sortilin to partition abnormally to the plasma membrane where it is normally not very abundant.

**Fig 5:**
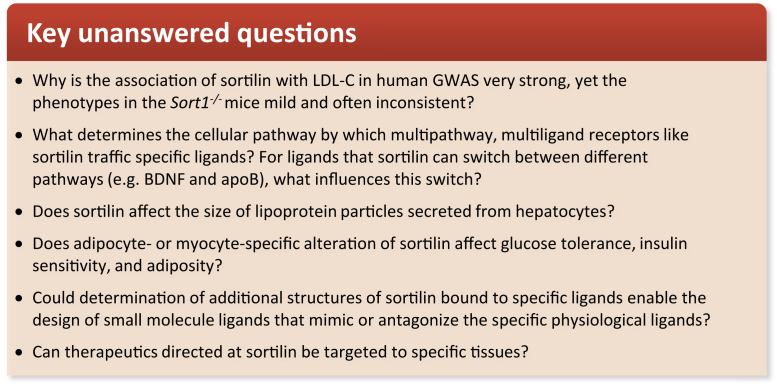
List of several key questions that remain about the function of sortilin and the role that it plays in metabolic disease.

We deliberately avoided reviewing sortilin’s role in neurological disease, where it is quite prominent. For example, it is a top-tier susceptibility gene for Alzheimer’s disease and frontotemporal dementia (21). The widespread involvement of sortilin in a range of physiological processes and human diseases, with many key questions yet to be answered, means that sortilin will continue to be the subject of intense investigation (Fig. 5).

## Conflict of interest

The authors declare that they have no conflicts of interest with the contents of this article.
